# A Multifunctional Fe-EGCG@RSL3 Nanomedicine Synergizes Ferroptosis Induction and Tumor Microenvironment Remodeling for Enhanced Bladder Cancer Immunotherapy

**DOI:** 10.34133/research.0735

**Published:** 2025-06-17

**Authors:** Chengjunyu Zhang, Sen Liu, Jianhui Zhang, Junlin Lu, Zehua Chen, Bolin Pan, Chu Liu, Ming Huang, Hengji Zhan, Hongjin Wang, Siting Chen, Kaiwen Jie, Baoqing He, Jingdie Wu, Ye Li, Haifeng Wang, Jing Zhao, Qiang Zhang, Xu Chen

**Affiliations:** ^1^ Department of Urology, Sun Yat-sen Memorial Hospital, Sun Yat-sen University, Guangzhou, China.; ^2^ Guangdong Provincial Key Laboratory of Malignant Tumor Epigenetics and Gene Regulation, Sun Yat-Sen Memorial Hospital, Sun Yat-Sen University, Guangzhou, China.; ^3^ Department of Urology, The Second Affiliated Hospital of Kunming Medical University, Kunming, China.; ^4^ Scientific Research Center, The Seventh Affiliated Hospital of Sun Yat-Sen University, Shenzhen, China.

## Abstract

Ferroptosis has promising potential for augmenting antitumor effects, but monotherapy with ferroptosis inducers in vivo has been reported to have limited efficacy in tumor management. The development of synergistic strategies with targeted capabilities is crucial for enhancing the antitumor efficacy of ferroptosis inducers. In this study, we designed and characterized a novel self-assembled nanomedicine by mixing ferrous ions (Fe^2+^) and epigallocatechin gallate (EGCG) in a controllable manner and encapsulating the ferroptosis inducer RSL3, named Fe-EGCG@RSL3. This multifunctional nanomedicine effectively induces ferroptosis and growth inhibition in bladder cancer cells and patient-derived organoids. In vivo, Fe-EGCG@RSL3 was enriched in the subcutaneous tumors of allogenic and xenograft mouse models, thereby substantially overcoming RSL3 resistance. Intravesical instillation of Fe-EGCG@RSL3 controls orthotopic bladder tumor progression. Furthermore, nanomedicine potentiates the therapeutic effect of anti-programmed cell death protein 1 (PD1) immunotherapy by increasing the cytotoxicity of CD8^+^ T cells to cancer cells and modulating the proportions of both T-cell and myeloid cell subpopulations within the tumor immune microenvironment. Overall, Fe-EGCG@RSL3 has dual functions as a multifaceted nanomedicine that integrates ferroptosis induction with immunomodulation, offering a novel and clinically translatable strategy for bladder cancer therapy.

## Introduction

Bladder cancer (BCa), the fourth leading cause of cancer in men, accounts for approximately 90% of cases of urothelial carcinoma [1]. Radical cystectomy (RC) combined with chemotherapy or immunotherapy is the first-line treatment for BCa [2]. Although chemotherapy or immunotherapy improves survival to some extent, a subset of patients still exhibits poor responses to either of these therapies, leading to missed opportunities for RC and reduced survival rates [3,4]. All these traditional systemic treatment strategies aim to induce cell death, predominantly apoptosis, a process through which cancer cells often develop resistance [5–7]. This necessitates the exploration of alternative types of cell death, which may provide new insights into overcoming resistance to conventional therapeutic modalities.

Ferroptosis, an iron-dependent and lipid peroxidation-characterized form of regulated cell death, has recently been identified as an emerging frontier in antitumor therapy [8]. Unlike conventional cell death patterns, which involve unique cell signaling cascades triggered by cell death executioner proteins, ferroptosis is initiated by the accumulation of oxidized phospholipids as a consequence of dysregulated lipid-redox metabolism [9]. Thus, ferroptosis-inducing therapy has promising potential for augmenting antitumor effects, especially in overcoming resistance to apoptosis-inducing therapies. Since the discovery of ferroptosis, ferroptosis inducers have shown dramatic cytotoxic effects on various cancer cells in vitro [8], including BCa cells [10]. Nevertheless, the inherent ferroptosis resistance of tumors renders the occurrence of ferroptosis relatively rare in vivo, even when conventional ferroptosis inducers are applied. Resistance to ferroptosis inducers in vivo has been demonstrated in colon cancer and T-cell leukemia [11]. In BCa xenograft models, monotherapy with ferroptosis inducers such as RSL3, IKE, and ML162 has been reported to exhibit suboptimal efficacy, which could be further improved via combination therapy [12]. This unsatisfactory antitumor efficacy may be partly attributed to the low solubility and unsatisfactory pharmacokinetics of most ferroptosis inducers. Furthermore, the nonspecific targeting ability of conventional ferroptosis inducers also limits their effects, as they may inadvertently kill antitumor immune cells in addition to tumor cells. Therefore, enhancing the therapeutic efficacy of ferroptosis inducers by increasing delivery efficiency and targeting capability is crucial for the development of ferroptosis-related antitumor strategies.

Nanoparticle-based drug delivery systems have demonstrated significant potential in antitumor therapy. Nanoparticles are natural or synthetic colloidal carriers with diameters ranging from 1 to 1,000 nm in size [13]. The advantages of nanoparticle-based delivery systems in antitumor therapy include improving drug solubility and penetration to the focus of tumors, as well as reducing drug dosage and toxicity [14]. Thus, nanoparticle-based drug delivery systems possess great potential to overcome the aforementioned barriers that limit the efficacy of ferroptosis inducers. Among the raw materials utilized in nanomedicine, polyphenols possess several advantages, especially in ferroptosis-inducing scenarios. First, polyphenols can coordinate with metal ions to form metal–phenolic networks. This facilitates the incorporation of ferrous ions (Fe^2+^), which are critical in the Fenton reaction to trigger ferroptosis, into the fundamental structure of nanomedicine to deliver exogenous Fe^2+^ into tumor cells. Second, polyphenol-derived metal–phenolic networks possess distinctive pH-responsive abilities, which facilitate the loading and controlled release of small-molecule drugs, potentially ferroptosis inducers, at tumor sites [15]. Third, polyphenols have been shown to modulate reactive oxygen species (ROS), which are another critical trigger of the ferroptosis-promoting Fenton reaction, to overcome cancer drug resistance [15]. Therefore, we aimed to construct a polyphenol-based multifunctional nanomedicine for the encapsulation and targeted delivery of ferroptosis inducers into tumor sites, thereby potentiating their antitumor effects.

**Fig. 1.**
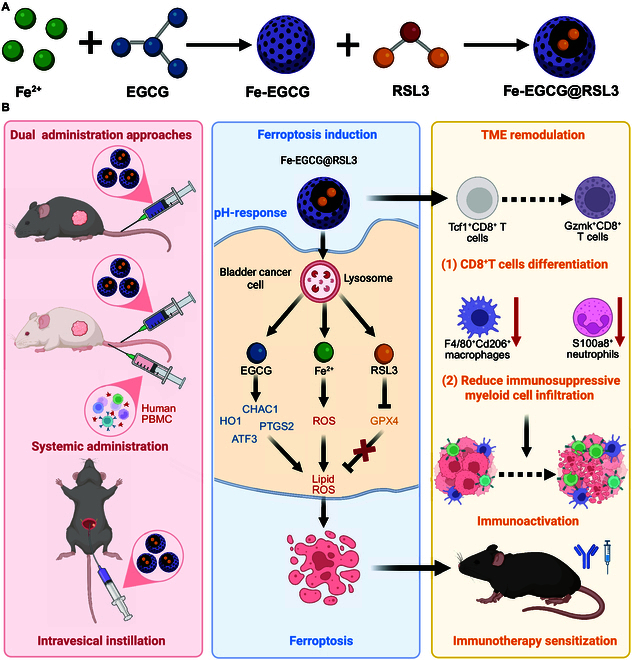
Fe-EGCG@RSL3 synergizes ferroptosis induction and tumor microenvironment remodeling for enhanced bladder cancer immunotherapy. (A) A schematic illustration of the preparation of nanovehicle Fe-EGCG and nanomedicine Fe-EGCG@RSL3. (B) The Fe-EGCG@RSL3 nanomedicine supports multiple administration routes (intravenous and intravesical), triggering ferroptosis in bladder cancer cells through multilevel mechanisms: delivering Fe^2+^, inducing ROS up-regulation and up-regulating ferroptosis-related genes. Beyond direct tumor killing, it remodels the immune microenvironment by promoting naive CD8^+^ T-cell differentiation into effector memory phenotypes and reducing immunosuppressive M2 macrophages and neutrophils, significantly sensitizing anti-PD1 therapy.

In our study, we exploited a novel polyphenol-derived nanomedicine that encapsulates RSL3, named Fe-EGCG@RSL3. The nanomedicine effectively delivers Fe^2+^ and RSL3 into BCa cells and patient-derived tumor organoids to induce ferroptosis. The ferroptosis-inducing and tumor-controlling effects of Fe-EGCG@RSL3 via systemic and intravesical administration were evaluated in both subcutaneous and orthotopic mouse models. Moreover, we investigated the immunotherapy-promoting efficacy of Fe-EGCG@RSL3 because of its ability to modulate the tumor immune microenvironment, as determined by single-cell RNA sequencing (scRNA-seq). Overall, our data indicate that Fe-EGCG@RSL3 has the potential to be an innovative ferroptosis-inducing antitumor strategy for BCa management (Fig. 1).

## Results

### Synthesis and characterization of the Fe-EGCG@RSL3 nanomedicine

**Fig. 2.**
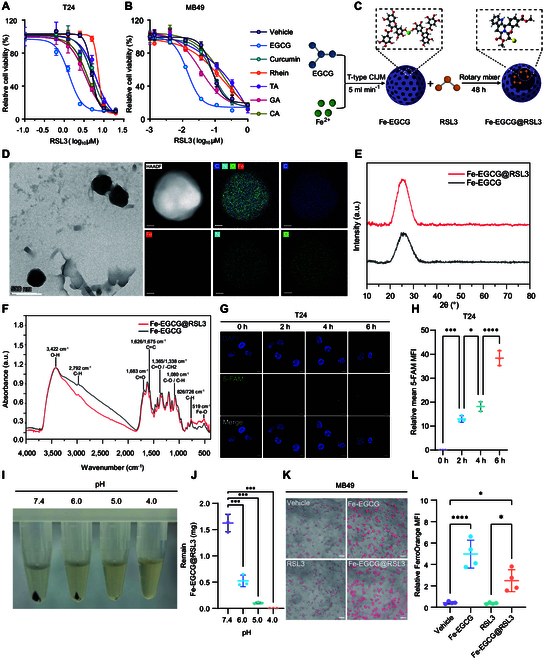
Material selection, synthesis, and characterization of the nanomedicine. (A and B) Cell viability of T24 (A) and MB49 (B) cells treated with RSL3 along with vehicle, EGCG, curcumin, rhein, TA, GA, or CA for 16 h. (C) Skeleton diagram of the synthesis of Fe-EGCG@RSL3. (D) TEM images of Fe-EGCG@RSL3; Elements representation: C, carbon; Fe, iron; O, oxygen; H, hydrogen; Cl, chlorine. Scale bar: 500 nm (left panel), 50 nm (right panel). (E) XRD analysis of the Fe-EGCG and Fe-EGCG@RSL3 nanomedicine. (F) FTIR spectrum of the Fe-EGCG and Fe-EGCG@RSL3 nanomedicine. (G and H) Confocal fluorescent images (G) and quantification analysis (H) showing the cellular uptake of 5-FAM-labeled Fe-EGCG (green) in T24 cells at 2, 4, and 6 h. The blue fluorescence represents the nucleus (DAPI). Scale bar: 10 μm. (I and J) Degradation of Fe-EGCG@RSL3 in PBS at different pH values. (K and L) Fluorescence images showing Fe^2+^ uptake after treatment with DMSO, RSL3 (1 μM), Fe-EGCG (46.6 μg ml^−1^), or Fe-EGCG@RSL3 (46.6 μg ml^−1^) for 3 h. Scale bar: 50 μm. The data are presented as the means ± SDs. Statistical significance was assessed via 2-tailed *t* tests and one-way ANOVA (ns *P* > 0.05; *P < 0.05; ***P < 0.001; **** *P <* 0.0001).

To screen the optimal carrier of nanomedicine for ferroptosis sensitization, polyphenols, which have been reported to exert antitumor effects, including epigallocatechin gallate (EGCG), curcumin, rhein, tannic acid (TA), gallic acid (GA), and caffeic acid (CA), have been adopted. Among these polyphenols, EGCG, along with RSL3, a ferroptosis inducer, has the greatest proferroptotic effect on both T24 (human BCa cell line, Fig. 2A) and MB49 (mouse BCa cell line, Fig. 2B) cells. To further test the safety of the indicated polyphenols, the proferroptotic effect on SV-HUC-1 cells (a normal human urothelium cell line) was evaluated. Unlike in tumor cells, none of the polyphenols contributed to the proferroptotic effect of RSL3 in SV-HUC-1 cells (Fig. S1). Next, we verified the changes in the ROS levels of the T24 and MB49 cell lines induced by the polyphenol EGCG. We found that, compared with other polyphenols, EGCG can effectively increase the ROS levels of BCa cells (Fig. S2).

On the basis of its ability to induce cell death in tumor cells and low cytotoxicity in normal urothelium, we selected EGCG to develop a nanomedicine together with ferrous ions (Fe^2+^), a ferroptosis initiator, via the flash nanocomplexation technique, named Fe-EGCG. To encapsulate RSL3, RSL3 was incubated with the prepared Fe-EGCG under sonication at 4 °C for 60 min and further incubated under a rotator for 48 h. The resulting Fe-EGCG@RSL3 nanomedicine was collected by absolute ethanol washes and centrifugation (Fig. 2C).

The physicochemical properties of the nanomedicine were first determined. As shown by transmission electron microscopy (TEM), the as-prepared Fe-EGCG has an amorphous spherical shape with an average size of ~200 nm with the expected elements of C, O, and Fe from EGCG and Cl and N from RSL3 (Fig. 2D), indicating the successful construction of Fe-EGCG and its loading of RSL3. X-ray diffraction (XRD) revealed the presence of completely amorphous peaks in the range of 20° to 30°, suggesting the amorphous structure of Fe-EGCG@RSL3 (Fig. 2E). As shown by Fourier transform infrared spectroscopy (FTIR), the characteristic absorption peak of the Fe-O stretching vibration appears at 516 cm^−1^, indicating that the phenol hydroxyl group of EGCG coordinates with Fe^2+^ to form the Fe-EGCG complex (Fig. 2F). Compared with those of Fe-EGCG, the absorption peaks of Fe-EGCG@RSL3 shifted in the range of 3,300 to 3,500 cm^−1^ and 1,650 to 1,700 cm^−1^ due to the specific amino and carbonyl groups in RSL3 (Fig. 2F). Furthermore, the peak shape of the O-H absorption peak at 3,422 cm^−1^ changes, indicating that RSL3 is loaded onto the surface of the Fe-EGCG complex mainly through hydrogen bonding or hydrophobic interactions (Fig. 2F). In summary, the nanovector Fe-EGCG was successfully constructed and loaded with the ferroptosis inducer RSL3 to form the nanomedicine Fe-EGCG@RSL3.

To investigate the cellular uptake of the nanomedicine, Fe-EGCG was labeled with 5-FAM and incubated with BCa cells. Confocal fluorescence imaging revealed that 5-FAM-Fe-EGCG was taken up in a time-dependent manner (Fig. 2G and H). TEM also revealed significant uptake and degradation of Fe-EGCG in the lysosomes of T24 cells (Fig. S3). Considering that the lysosome is an acidic organelle, we then tested the degradation of the nanomedicine at different pH values. Fe-EGCG degrades under acidic conditions (pH 4.0–6.0, Fig. 2I and J), which is similar to what occurs in the tumor microenvironment and lysosomes. FerroOrange, a ferrous ion fluorescent probe, increased the intracellular ferrous ion concentration after Fe-EGCG was incubated with cancer cells (Fig. 2K and L and Fig. S4). Additionally, the delivery efficiency of Fe^2+^ by Fe-EGCG is also greater than that of free Fe^2+^ at the same concentration (Fig. S5). These results demonstrated that the nanomedicine of Fe-EGCG could be taken up by tumor cells and released in acidic environments, increasing the intracellular ferrous concentration.

### Ferroptosis-promoting effect of Fe-EGCG@RSL3 in vitro

**Fig. 3.**
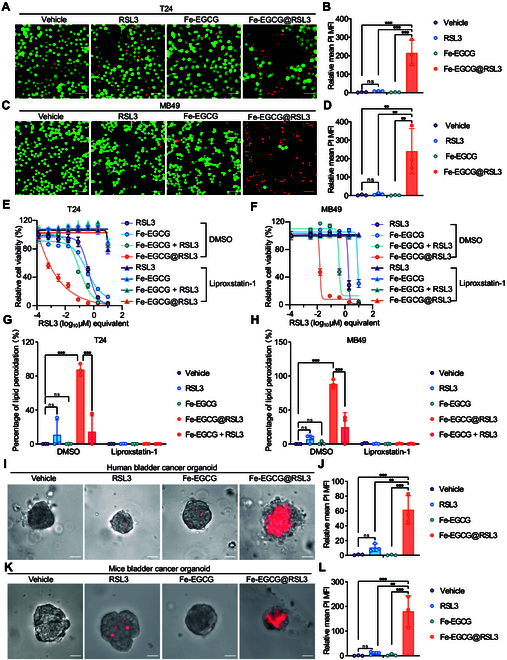
The nanomedicine strongly induces ferroptosis in vitro*.* (A to D) Representative fluorescence images of T24 (A) and MB49 (C) cells with indicating treatment stained with Calcein-AM (green, viable) and PI (red, dead). Scale bar: 20 μm. Quantification of the relative mean PI MFI in T24 (B) and MB49 (D) cells. (E and F) Cell viability of T24 (E) and MB49 (F) cells treated with RSL3 (1 μM), Fe-EGCG (44.6 μg ml^−1^), Fe-EGCG@RSL3 (44.6 μg ml^−1^), and Fe-EGCG + RSL3 (along with DMSO or liproxstatin-1) for 16 h. (G and H) Cell peroxidation measurement of T24 (G) and MB49 (H) cells treated with RSL3 (1 μM), Fe-EGCG (44.6 μg ml^−1^), Fe-EGCG@RSL3 (44.6 μg ml^−1^), and Fe-EGCG + RSL3 (along with DMSO or liproxstatin-1) for 3 h. (I and J) Representative immunofluorescence images (I) and quantification (J) of dead cells stained with PI in human BCa PDOs treated with vehicle, RSL3 (1 μM), Fe-EGCG (44.6 μg ml^−1^), or Fe-EGCG@RSL3 (44.6 μg ml^−1^) for 8 h. Scale bar: 20 μm. (K and L) Representative immunofluorescence images (K) and quantification (L) of dead cells stained with PI in mouse BCa organoids treated with vehicle, RSL3 (1 μM), Fe-EGCG (44.6 μg ml^−1^), or Fe-EGCG@RSL3 (44.6 μg ml^−1^) for 6 h. Scale bar: 20 μm. The data are presented as the means ± SDs. Statistical significance was determined using 2-tailed *t* tests and one-way ANOVA. (ns, *P* > 0.05; **P < 0.01; ***P < 0.001).

The cytotoxic effects of Fe-EGCG and Fe-EGCG@RSL3 were evaluated. As shown by the Calcein acetoxymethyl (AM)/propidium iodide (PI) staining results, Fe-EGCG alone had no cytotoxic effect at a dose of 1 μM. Fe-EGCG@RSL3 significantly promoted cell death when RSL3 was encapsulated at a low concentration, which resulted in no cytotoxicity alone within 4 h (Fig. 3A to D). To determine whether the cytotoxic effect of Fe-EGCG@RSL3 is predominantly attributed to ferroptosis in various BCa cell lines, including T24, UM-UC-3, RT112, 5637, and MB49 cell lines, CCK8 assays were also performed. A similar effect of the nanomedicine was confirmed. Furthermore, the cytotoxicity of Fe-EGCG@RSL3 could be rescued by the ferroptosis inhibitor liproxstatin-1, indicating the ferroptosis-specific cytotoxicity of the nanomedicine (Fig. 3E and F and Figs. S6 and S7). We also confirmed that Fe-EGCG@RSL3 was no harm to SV-HUC-1 cells under therapeutic doses (Fig. S8). As critical characteristics of ferroptosis, lipid peroxidation and ROS induction were slightly elevated when cancer cells were treated with Fe-EGCG along with RSL3 and dramatically increased when they were treated with Fe-EGCG@RSL3 (Fig. 3G and H and Fig. S9).

To further determine the ferroptosis-promoting effect of Fe-EGCG@RSL3 in a clinical specimen-based model, we constructed patient-derived organoids (PDOs) of BCa. Under sublethal concentrations of RSL3 and Fe-EGCG monotherapy, Fe-EGCG@RSL3 significantly induced cell death in the PDOs of BCa patients (Fig. 3I and J). We also established mouse BCa organoids to investigate the cytotoxic effect of Fe-EGCG@RSL3 in mouse-derived samples. Similarly, we also observed a significant killing effect of Fe-EGCG@RSL3 in mouse BCa organoids (Fig. 3K and L). These results indicate that Fe-EGCG@RSL3 has cytotoxic effects not only in both cancer cell lines but also in human- and mouse-derived tumor specimen-based models, indicating its translational potential for clinical application.

### Ferroptosis-promoting mechanism of Fe-EGCG@RSL3

**Fig. 4.**
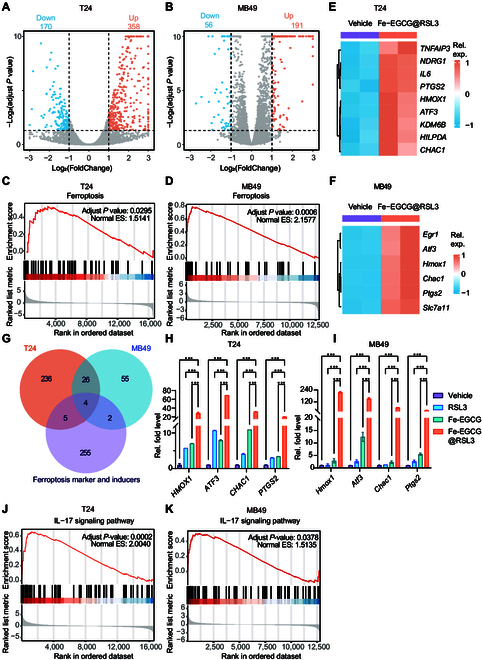
The nanomedicine up-regulated the transcriptional levels of ferroptosis-related genes in BCa. (A and B) Volcano plot of gene expression changes in T24 cells (A) and MB49 cells (B) treated with vehicle or Fe-EGCG@RSL3 (44.6 μg ml^−1^) for 4 h. (C and D) GSEA enrichment analysis of ferroptosis-related genes from the transcriptome data of T24 (C) or MB49 (D) cells treated with vehicle or Fe-EGCG@RSL3. (E and F) Heatmap of ferroptosis-related gene changes in T24 cells treated with vehicle or Fe-EGCG@RSL3 for 4 h. (G) Venn diagram showing the overlapping genes among ferroptosis-related genes, up-regulated genes with LogFC > 1.5 in T24 cells, and up-regulated genes with LogFC > 1.5 in MB49 cells. (H and I) qRT-PCR analysis of *HMOX-1*, *ATF3*, *CHAC1*, and *PTGS2* mRNAs in T24 cells (H) and *Hmox-1*, *Atf3*, *Chac1*, and *Ptgs2* mRNAs in MB49 cells (I) treated with vehicle, RSL3 (1 μM), Fe-EGCG (44.6 μg ml^−1^), or Fe-EGCG@RSL3 (44.6 μg ml^−1^) for 4 h. (J and K) GSEA enrichment analysis of the IL-17 signaling pathway from the transcriptome data of T24 (J) or MB49 (K) cells treated with vehicle or Fe-EGCG@RSL3. The data are presented as the means ± SDs. Statistical significance was determined using one-way ANOVA (***P < 0.001).

To investigate how Fe-EGCG@RSL3 influences BCa cells to promote ferroptosis, vehicle- or Fe-EGCG@RSL3-treated BCa cells were collected for transcriptome analysis. In T24 cells, 358 up-regulated genes and 170 down-regulated genes were identified (Fig. 4A). In MB49 cells, 191 up-regulated genes and 56 down-regulated genes were identified (Fig. 4B). The ferroptosis gene set was significantly enriched in Fe-EGCG@RSL3-treated tumor cells (Fig. 4C and D), indicating that the up-regulation of ferroptosis-related genes contributes to the ferroptosis-promoting mechanism of the nanomedicine. To further investigate the specific gene alterations that contribute to the ferroptosis-promoting effect of the nanomedicine, ferroptosis-related genes among the genes whose log_2_FC > 1.5 in T24 and MB49 cells were identified, as shown in heatmaps (Fig. 4E and F). We subsequently identified 4 key genes that are dysregulated in both Fe-EGCG@RSL3-treated MB49 and T24 cells (Fig. 4G), including *ATF3* (*Atf3*), *HMOX1* (*Hmox1*), *CHAC1* (*Chac1*), and *PTGS2* (*Ptgs2*). The expression of these genes was verified via real-time quantitative polymerase chain reaction (RT-qPCR). The expression of these genes was dramatically elevated in T24 and MB49 cells upon treatment with Fe-EGCG@RSL3 compared with the vehicle control, RSL3, or Fe-EGCG (Fig. 4H and I). Furthermore, we evaluated the protein expression levels of these ferroptosis markers as our nanomedicine is applied to MB49 and T24 cells after Fe-EGCG@RSL3 treatment. Consistent with mRNA expression, ATF3, HO1, CHAC1, and PTGS2 are significantly up-regulated in cancer cells after challenging with Fe-EGCG@RSL3 (Fig. S10).

*ATF3* promotes ferroptosis by suppressing the system Xc^−^ transporter and thereby decreasing the intracellular level of the lipid peroxidation detoxifier GSH [16]. *ATF3* was up-regulated by Fe-EGCG, and its expression increased more than 100-fold under treatment with Fe-EGCG@RSL3. The up-regulation of HO-1, encoded by *Hmox1*, by Fe-EGCG@RSL3 might increase the labile iron pool, which contributes to lipid peroxidation [17,18]. Stimulation by *CHAC1* may also sensitize cells to ferroptosis by regulating GSH homeostasis [19]. *PTGS2*, encoding COX-2, is a well-documented ferroptosis-initiating marker. Overall, these dysregulated genes strongly influence the proferroptosis mechanisms of nanomedicine and highlight substantial alterations in ferroptosis-related cell status. Moreover, gene set enrichment analysis (GSEA) revealed enrichment of the interleukin-17 (IL-17) signaling pathway (Fig. 4J and K). High expression of IL-17 may result in superior responsiveness to PD1 therapy [20], suggesting a potential immunomodulatory effect in Fe-EGCG@RSL3-treated tumor cells.

### Fe-EGCG@RSL3 promotes ferroptosis and sensitizes BCa cells to immunotherapy in vivo

We investigated whether the Fe-EGCG nanomedicine could accumulate specifically in tumors. We established an MB49/luciferase (MB49/luc) subcutaneous tumor model in C57BL/6 mice. Free DiR and Fe-EGCG-encapsulated DiR were then administered via tail vein injection. Afterwards, the DiR distribution was evaluated via an in vivo imaging system (IVIS). As shown in Fig. S11, both DiR- and Fe-EGCG-encapsulated DiR predominantly accumulated in the liver and spleen, with minor accumulation in the lung. Notably, Fe-EGCG-encapsulated DiR was significantly enriched at the tumor site and remained detectable in the tumor for at least 3 days. These results indicate that the Fe-EGCG nanomedicine is capable of tumor targeting.

**Fig. 5.**
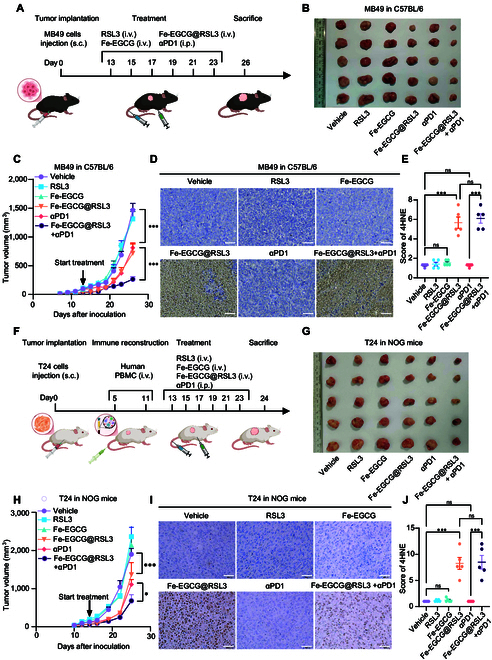
The nanomedicine induces ferroptosis and sensitizes BCa cells to immunotherapy in vivo*.* (A) Schematic illustration of the treatment schedule for C57BL/6 mice. The mice were randomly separated (*n* = 5) and separately treated with vehicle, RSL3 (10 mg kg^−1^), Fe-EGCG (76.6 mg kg^−1^), Fe-EGCG@RSL3 (76.6 mg kg^−1^), αPD1 (10 mg kg^−1^), or Fe-EGCG@RSL3 + αPD1. (B) Tumors harvested from tumor-bearing C57BL/6 mice after different treatments on day 26. (C) Tumor volume in MB49 (*n* = 5) tumor-bearing C57BL/6 mice treated with vehicle, RSL3 (10 mg kg^−1^), Fe-EGCG (76.6 mg kg^−1^), Fe-EGCG@RSL3 (76.6 mg kg^−1^), αPD1 (10 mg kg^−1^), or Fe-EGCG@RSL3 + αPD1. (D and E) Representative IHC staining for 4HNE in tumors harvested from MB49 tumor-bearing C57BL/6 mice. Scale bar: 500 μm (D). Quantification (E) of 4HNE expression in tumors. (F) Schematic illustration of the treatment schedule for NOG-SCID mice. A total of 1×10^8^ activated PBMC cells were injected into the mice through the tail vein to restore T-cell immunity. The mice were then randomly separated (*n* = 5) and separately treated with vehicle, RSL3 (10 mg kg^−1^), Fe-EGCG (76.6 mg kg^−1^), Fe-EGCG@RSL3 (76.6 mg kg^−1^), αPD1 (10 mg kg^−1^), or Fe-EGCG@RSL3 + αPD1. (G) Tumors harvested from tumor-bearing C57BL/6 mice after different treatments on day 24. (H) Tumor growth in T24 (*n* = 5) tumor-bearing NOG-SCID mice treated with vehicle, RSL3 (10 mg kg^−1^), Fe-EGCG (76.6 mg kg^−1^), Fe-EGCG@RSL3 (76.6 mg kg^−1^), αPD1 (10 mg kg^−1^), or Fe-EGCG@RSL3 + αPD1. (I and J) Representative images of IHC staining for 4HNE in tumors harvested from T24 tumor-bearing NOG-SCID mice. Scale bar: 500 μm (I). Quantification (J) of 4HNE expression in tumors. The data are presented as the means ± SDs (H and J) or means ± SEMs (D and F). Statistical significance was determined via 2-tailed *t* tests and 2-way ANOVA (ns, *P* > 0.05; *P < 0.05; ***P < 0.001).

Since both ferroptosis and EGCG have been demonstrated to significantly modulate antitumor immunity, we subsequently tested the therapeutic efficacy and immunotherapeutic sensitization effects of intravenous administration of Fe-EGCG and Fe-EGCG@RSL3 in 2 distinct subcutaneous tumor models: the MB49 allograft model (Fig. 5A) and the T24 xenograft model with peripheral blood mononuclear cell (PBMC) immune reconstruction (Fig. 5F). In both models, RSL3 monotherapy and Fe-EGCG monotherapy had no significant antitumor effects. Compared with anti-PD1 immunotherapy, Fe-EGCG@RSL3 had a comparable therapeutic effect, while the combination of Fe-EGCG@RSL3 and an anti-PD-1 agent had the best antitumor effect (Fig. 5B, C, G, and H). These results demonstrate that Fe-EGCG@RSL3 has an antitumor effect and promotes the efficacy of immunotherapy. To further confirm the ferroptosis-promoting effect of 4-hydroxynonenal (4HNE), a lipid peroxidation by-product, in vivo, we performed immunohistochemistry (IHC) staining to visualize ferroptosis regions in tumor samples. In both mouse models, Fe-EGCG@RSL3 significantly induced the production of 4HNE, regardless of whether it was treated with anti-PD1 or administered alone. However, Fe-EGCG alone and RSL3 monotherapy rarely elicited 4HNE production (Fig. 5D, E, I, and J). These results indicate that Fe-EGCG@RSL3 induces ferroptosis in vivo.

To evaluate the safety of the nanomedicine, we harvested the liver, heart, brain, lungs, and kidneys of the treated mice. Hematoxylin and eosin (H&E) staining revealed that Fe-EGCG@RSL3 treatment did not obviously alter the morphology of these essential organs, although Fe-EGCG was significantly enriched in the liver, spleen, and lung (Fig. S12). Serum samples were collected from the mice for biochemical tests. The liver and renal dysfunction-related marker levels were within normal ranges (Fig. S12). The hemolysis experiment indicated that the nanomedicine had no significant effect on red blood cells at therapeutic concentrations during systemic administration (Fig. S13). Taken together, these results suggest that normal organs and tissues do not harm this nanomedicine-based ferroptosis-inducing therapy.

**Fig. 6.**
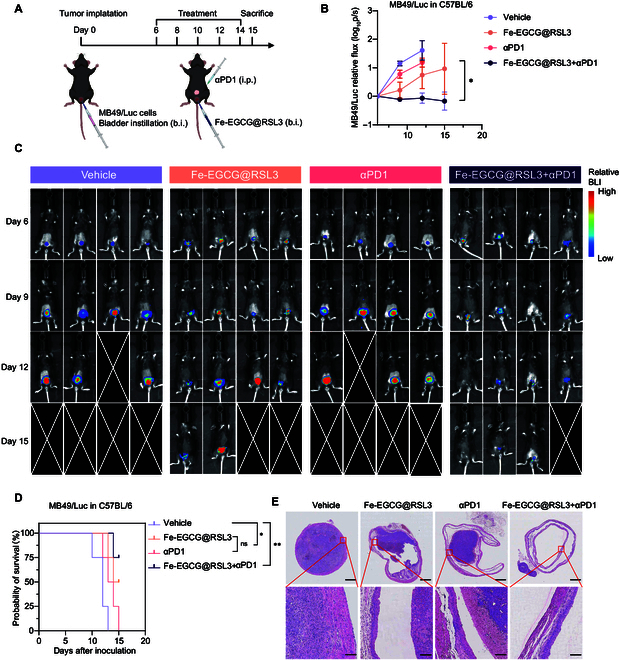
Intravesical instillation of the nanomedicine combined with immunotherapy exhibit better tumor control in BCa orthotopic model. (A) Schematic illustration of the treatment schedule for an orthotopic model of BCa in C57BL/6 mice. The mice were randomly separated (*n* = 4 per group) and were separately treated with vehicle, Fe-EGCG@RSL3 (50 μl of 76.6 mg kg^−1^), αPD1 (10 mg kg^−1^), or Fe-EGCG@RSL3 + αPD1. (B and C) Relative fluorescence intensity (B) and IVIS bioluminescence images (C) of different groups receiving the indicated treatments. (D) Kaplan–Meier survival curve of MB49/luc orthotopic tumor-bearing mice receiving the indicated treatments. (E) H&E staining of tumors harvested at the endpoint from individual mice in different groups. Scale bars: 1 mm (top) and 100 μm (bottom). The data are presented as the means ± SEMs. Statistical significance was assessed via 2-way ANOVA and the log-rank test (*P < 0.05; **P < 0.01).

Given that the bladder is an organ that interfaces with the external environment, intravesical administration offers a strategy with better accessibility to cancer lesions and enhanced safety. Thus, we evaluated the efficacy of intravesical instillation of the nanomedicine in an orthotopic BCa model in C57BL/6 mice (Fig. 6A). In this orthotopic model, systemic anti-PD1 therapy has unsatisfactory antitumor efficacy. Fe-EGCG@RSL3 has a tumor-control effect, and combining intravesical Fe-EGCG@RSL3 therapy with systemic anti-PD1 therapy results in better control of orthotopic bladder tumors (Fig. 6B and C). The combination therapy significantly prolonged the survival of the tumor-bearing mice (Fig. 6D). H&E staining further confirmed the antitumor effect of the combination therapy, while the nanomedicine did not significantly damage normal urothelium cells. (Fig. 6E)*.* These findings suggest that our nanomedicine is capable of penetrating and accumulating in tumors under intravesical instillation conditions, thereby exerting ferroptosis-inducing and immunotherapy-promoting effects.

### Fe-EGCG@RSL3 recapitalizes T-cell subpopulations to promote immunotherapy

**Fig. 7.**
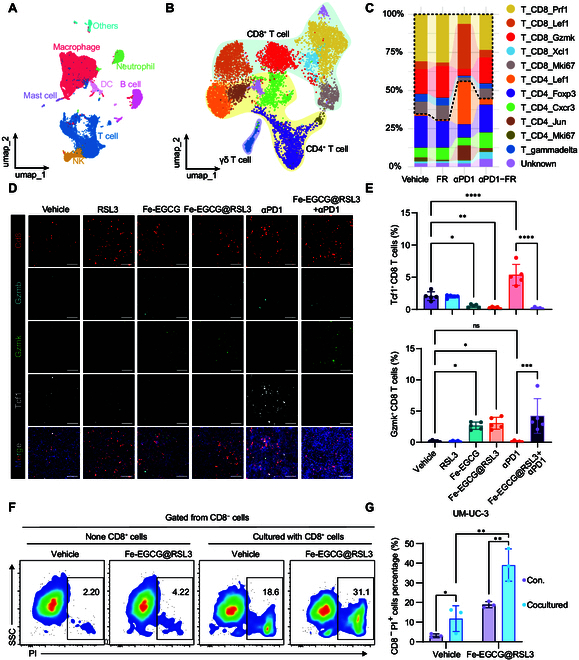
The nanomedicine recapitalizes T-cell subpopulations that contribute to the immunotherapy-promoting effect. (A) Transcriptional landscapes of CD45^+^ single-cell populations (*n* = 31,588) across 4 experimental cohorts, vehicle control, Fe-EGCG@RSL3 monotherapy (FR cohort), anti-PD1 monotherapy (αPD1 cohort), and Fe-EGCG@RSL3 combined with anti-PD1 (αPD1-FR cohort), were subjected to dimensionality reduction analysis via uniform manifold approximation and projection (UMAP) algorithms. Three mice from each group were used for analysis. Each dot represents a single cell, and the colors represent clusters denoted by inferred cell types. (B) UMAP embedding of single-cell transcriptional profiles from the T-cell subset. Each dot represents a single cell, and the colors represent clusters denoted by inferred cell types. (C) Relative proportions of the T-cell subset in each group. (D) Representative immunofluorescence images of CD8^+^ T cells and Gzmk^+^ CD8^+^ T cells, Gzmb^+^ CD8^+^ T cells, and Tcf1^+^ CD8^+^ T infiltration in tumors harvested from MB49 tumor-bearing C57BL/6 mice. Scale bar: 2.5 μm. (E) Quantitative analysis of Gzmk^+^ CD8^+^ T-cell and Tcf1^+^ CD8^+^ T-cell infiltration. (F and G) Flow cytometric dot plots and cell death indices of UM-UC-3 cells under CD8^+^ T-cell-depleted versus replete conditions. Quantitative results are expressed as mean ± standard deviation. The data are presented as the means ± SDs. Intergroup differences were determined by one-way ANOVA (ns, *P* > 0.05; *P < 0.05; **P < 0.01; ***P < 0.001; ****P < 0.0001).

Considering that nanomedicine enhances the efficacy of immunotherapy, we further investigated how Fe-EGCG@RSL3 induces alterations in the TME. scRNA-seq was conducted to compare the changes in CD45^+^-sorted immune cells among the vehicle, Fe-EGCG@RSL3 (FR group), anti-PD1 (αPD1 group), and Fe-EGCG@RSL3 combined with anti-PD1 (αPD1-FR group) treatment groups. The transcriptome data of 31,588 CD45^+^-sorted single cells from 3 mice in each group were obtained. Uniform manifold approximation and projection (UMAP) analysis of the expression data revealed that the single cells were primarily identified into 8 clusters, including macrophages/monocytes, neutrophils, dendritic cells, B cells, T cells, NK cells, mast cells, and other confounding cells (Fig. 7A). We identified these clusters on the basis of the expression of signature genes in various clusters. The density plots depict the expression levels of signature genes (Fig. S14).

In anti-PD1 immunotherapy, T cells, especially CD8^+^ T cells, are the predominant effectors. Thus, we initially analyzed T-cell heterogeneity, which was regulated by treatment with Fe-EGCG and Fe-EGCG@RSL3. We identified 5 CD8 clusters, 5 CD4 clusters, and a γδT cell cluster (Fig. 7B). The expression of the top 50 marker genes in each cluster is shown in the heatmap (Fig. S15). The proportion of CD8^+^ T cells was slightly greater in the FR and αPD1-FR groups than in the vehicle and αPD1 groups (Fig. 7C).

On the basis of the functional genes identified among the top marker genes (Fig. S15), we could preliminarily infer the potential functions of each T-cell cluster (Fig. 7C). The total CD8^+^ T cells were identified into the following 5 clusters. Among these CD8^+^ T-cell clusters, we observed a decrease in the proportion of cluster T_CD8_Lef1 and an increase in the proportion of T_CD8_Gzmk in both the FR group compared with the vehicle group and in the αPD1-FR group compared with the αPD1 group. According to the marker genes (Fig. S15), we annotated T_CD8_Lef1 as a naïve CD8 T-cell cluster and T_CD8_Gzmk as an effector memory CD8 T-cell cluster [21]. These findings suggest that Fe-EGCG@RSL3 may promote the differentiation of naïve CD8^+^ T cells, primarily into effector memory CD8^+^ T cells. Furthermore, T_CD8_Prf1 may exhibit traditional effector functions due to its expression of cytotoxic and exhaustion-related gene markers (Fig. S15). Thus, we calculated the gene expression score of the effector-related gene set in cells from cluster T_CD8_Prf1 among the groups. Effector-related gene scores were significantly greater in the αPD1-FR group than in the anti-PD1 group (Fig. S16). Moreover, the expression levels of *Pdcd1* in cells from the T_CD8_Prf1 cluster were also reduced as Fe-EGCG@RSL3 and anti-PD1 were administered in combination (Fig. S15). Therefore, Fe-EGCG@RSL3 may potentiate anti-PD1 therapeutic efficacy by promoting effector-related gene expression and reducing the expression of PD1 in cytotoxic CD8^+^ T cells.

To verify these findings, we first analyzed the proportions of CD8^+^ T cells and PD-1^+^ CD8^+^ T cells via flow cytometry. The combination of Fe-EGCG@RSL3 and anti-PD1 slightly promoted the infiltration of CD8^+^ T cells (Fig. S17). A significant decrease in Pd1^+^ CD8^+^ T cells was observed in the αPD1-FR group (Fig. S18), which is consistent with our findings from scRNA-seq. Next, multiplex immunofluorescence staining of Tcf1, Gzmk, and Gzmb was conducted to identify naïve T cells, effector memory T cells, and cytotoxic T cells in harvested mouse tumors. Fe-EGCG and Fe-EGCG@RSL3 treatment diminished the proportion of naïve CD8^+^ T cells and increased the infiltration of effector memory CD8^+^ T cells while having a minimal impact on the proportion of cytotoxic CD8^+^ T cells, indicating that Fe-EGCG has the potential to promote naïve CD8^+^ T-cell differentiation into effector memory CD8^+^ T cells. Furthermore, when Fe-EGCG@RSL3 was combined with anti-PD1 therapy, the population of naïve CD8^+^ T cells markedly decreased, whereas there was a significant increase in both effector memory and cytotoxic CD8^+^ T cells (Fig. 7D and E and Fig. S19). These results indicate that Fe-EGCG@RSL3 potentially promotes naïve CD8^+^ T-cell differentiation into effector and effector memory clusters, improving their cytotoxic function to potentiate immunotherapy.

On the basis of these findings, we further tested whether Fe-EGCG@RSL3 affects the ability of T cells to kill tumor cells via an in vitro T-cell cytotoxicity assay. On the basis of their insensitivity to ferroptosis (Fig. S20), we chose UM-UC-3 cells as the target cells for the T-cell-mediated cytotoxicity assay. The administration of Fe-EGCG@RSL3 to UM-UC-3 cells significantly enhanced CD8^+^ T-cell-mediated cytotoxicity in a synergistic manner (Fig. 7F and G). Overall, these results suggest that Fe-EGCG@RSL3 could potentiate the therapeutic effect of anti-PD1 immunotherapy by modifying the constitution and function of CD8^+^ T cells against cancer cells.

Additionally, in CD4^+^ T cells, the naïve cluster T_CD4_Lef1 was also diminished under Fe-EGCG@RSL3 therapy. Cluster T_CD4_Cxcr3, with high expression of Cxcr3 and Cd44, which can be characterized as T helper 1 (Th1) cells, was elevated under Fe-EGCG@RSL3 administration regardless of whether anti-PD1 was used. More Th1 cells may contribute to the activation of CD8 T cells to improve their cytotoxic function. In the T_CD4_Jun cluster, *Fos*, *Jun*, and *Nr4a1* are highly expressed, presenting a pattern that has been reported to be a stress response state [22]. The stress response of CD4^+^ T cells has been characterized as a dysfunctional state, contributing to immunotherapy resistance in addition to exhaustion [22], which is also decreased under Fe-EGCG@RSL3 administration (Fig. 7C). These results suggest that alterations in CD4^+^ T cells may also contribute to the immunotherapy-facilitating effect of nanomedicine.

### Fe-EGCG@RSL3 remodels myeloid immune cells within the tumor microenvironment

**Fig. 8.**
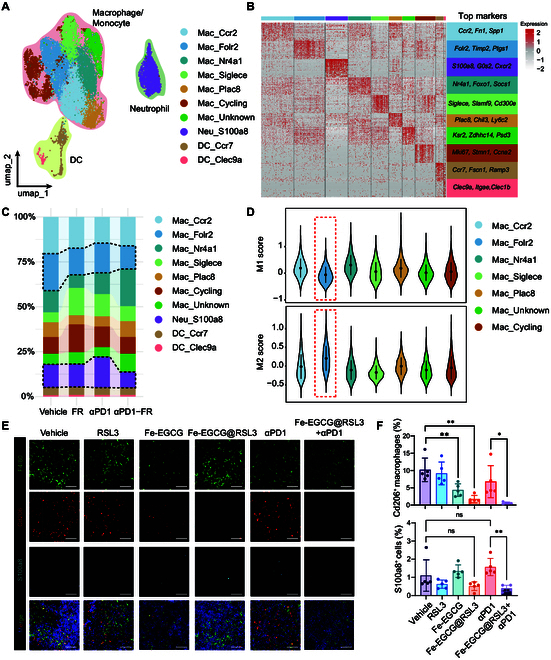
The nanomedicine remodels the macrophages and neutrophils in the tumor microenvironment. (A) Dimensionality reduction visualization via UMAP applied to single-cell transcriptomic datasets derived from myeloid lineage subsets (macrophages/monocytes, neutrophils, and dendritic cells). Discrete points correspond to individual cells, with cluster identities chromatically coded according to computationally annotated cellular phenotypes. (B) Heatmap of the top 50 marker genes of each macrophage/monocyte, neutrophil, and DC subset. (C) Relative proportions of the myeloid cell types in each group. (D) Violin plots showing the expression scores of M1 and M2 markers among different macrophage/monocyte clusters. (E) Representative immunofluorescence images of F4/80^+^ Cd206^+^ M2 macrophage and S100a8^+^ neutrophil infiltration in tumors harvested from MB49 tumor-bearing C57BL/6 mice. Scale bar: 2.5 μm. (F) Quantitative analysis of F4/80^+^ Cd206^+^ M2 macrophage and S100a8^+^ neutrophil infiltration. The data are presented as the means ± SDs. Intergroup differences were determined by one-way ANOVA (ns, *P* > 0.05; *P < 0.05; **P < 0.01).

The major components of myeloid cells, including macrophages/monocytes, neutrophils and DCs, were further analyzed. The macrophages/monocytes were classified into 7 different clusters on the basis of the differential expression of the identified marker genes (Fig. 8A and B). Among these clusters, the Mac_Folr2 cluster was reduced under Fe-EGCG@RSL3 treatment (Fig. 8C). To characterize this cluster, we calculated the expression scores of M1 and M2 markers [23] among all the macrophage/monocyte clusters. As shown in Fig. 8D, the Mac_Folr2 cluster presented the lowest expression score of M1 markers but the highest expression score of M2 markers. Therefore, the down-regulation of the Mac_Folr2 cluster, which is an M2-like macrophage with immunosuppressive properties, may have contributed to enhancing the immunotherapeutic efficacy of Fe-EGCG@RSL3. Neutrophils have been reported to attenuate immunotherapy, which is also diminished in the αPD1-FR group compared with the anti-PD1 monotherapy group (Fig. 8C). Moreover, DCs are also classified into 2 different clusters, although their proportions remain stable among different groups (Fig. 8C).

We further confirmed the differential distribution of the M2-like Mac_Folr2 cluster and neutrophils by staining for the macrophage marker F4/80, the M2 marker Cd206, and the neutrophil marker S100a8. The administration of Fe-EGCG or Fe-EGCG@RSL3 resulted in a decrease in the number of CD206^+^ M2-like macrophages. The population of S100a8^+^ neutrophils was lower in the combination therapy group than in the anti-PD1 monotherapy group (Fig. 8E and F). Overall, we demonstrated the tumor microenvironment-modulating effect of Fe-EGCG@RSL3, providing deeper insights into the mechanisms underlying the immunotherapy-enhancing phenomenon.

## Discussion

In this study, we reported a nanomedicine containing RSL3, EGCG, and Fe^2+^, termed Fe-EGCG@RSL3, designed to induce ferroptosis and increase the efficacy of immunotherapy in BCa. Upon accumulation in tumor lesions, the nanomedicine exhibits pH-responsive characteristics, facilitating its entry into cancer cells and subsequent release of RSL3 and Fe^2+^ to promote BCa ferroptosis. Moreover, the administration of Fe-EGCG@RSL3 remodeled the tumor microenvironment to sensitize BCa cells to anti-PD1-based immunotherapy. Mechanistically, Fe-EGCG@RSL3 promotes naïve T-cell differentiation, enhances the cytotoxic function of effector CD8^+^ T cells, increases the proportions of CD8^+^ effector memory T cells and Th1-like CD4^+^ T cells, and reduces the infiltration of stress response state CD4^+^ T cells and protumor Folr2-expressing M2-like macrophages. Overall, this innovative nanomedicine delivers RSL3 into tumors, providing a novel, safe, and efficient approach for the management of this disease.

Despite the growing interest in targeting ferroptosis as an antitumor therapy for years, the antitumor efficacy of ferroptosis-based regimens, particularly in immunocompetent mouse models, remains controversial. First, RSL3 and other known GPX4 inhibitors interact with the selenocysteine residue of GPX4, a key and frequently targetable protein that plays a dominant role in scavenging lipid peroxides. This interaction is mediated by their covalent reactive alkyl chloride group, which may lead to reduced selectivity and suboptimal pharmacokinetic profiles [24]. However, the molecular structure of GPX4 lacks deep-druggable cavities for small-molecule binding, which poses significant challenges in the development of novel targetable drugs. Furthermore, the systemic application of ferroptosis inducers may compromise the function or viability of immune cells that possess antitumor properties, even resulting in the promotion of tumor growth [11]. Consequently, ferroptosis-inducing therapy is often applied in combination with other treatments, primarily immunotherapy and chemotherapy, to overcome resistance to ferroptosis and achieve enhanced antitumor efficacy. Improving the targeted efficacy of ferroptosis inducers could address the limitations of ferroptosis monotherapy, thereby expanding its clinical application [25]. In light of the advantages of the polyphenol structure of EGCG, we developed a nanomedicine based on the coordination bond between EGCG and Fe^2+^ ions, as well as the hydrophobic interaction between EGCG and RSL3.

Delivering Fe^2+^ or regulating redox balance within cancer cells are common strategies to potentiate ferroptosis. Compared with other metal ions, such as Cu^+^ or Mn^2+^, Fe^2+^ has a higher catalytic efficiency and is still effective for low concentrations of H_2_O_2_, overcoming the limitation of insufficient H_2_O_2_ in the tumor microenvironment [26]. However, because Fe^2+^ is generally oxidized in the internal environment and has a limited uptake rate due to its low transferrin combination ability, the direct application of Fe^2+^ is ineffective in inducing tumor ferroptosis [27,28]. Our nanomedicine demonstrated high efficiency in terms of Fe^2+^ delivery and improved the stability of Fe^2+^, enabling it to more effectively induce the Fenton reaction in tumor tissues and exhibit a better synergistic effect in inducing ferroptosis*.* Through screening among the bioactive polyphenols, we identified the ferroptosis-promoting effect of EGCG in BCa. EGCG, as one of the main active components of natural catechins, has been widely studied in the field of tumor prevention and treatment for years. Although EGCG has distinct antitumor effects in vitro, its poor pharmacokinetics are a major problem for its clinical application. Specifically, the high polarity of EGCG leads to low oral absorption efficiency. Furthermore, EGCG undergoes rapid autooxidation and is highly unstable in a neutral internal environment when it is administered systemically [29]. The polyphenol hydroxyl groups of EGCG enable it to form macromolecular polymers through covalent or noncovalent interactions with various compounds, providing a foundation for its use as a nanomedicine matrix [30]. By forming nanomedicines, the in vivo stability of EGCG could be improved. Moreover, nanomedicine-formed EGCG may penetrate and become enriched in tumor lesions, reducing the possibility of liver and kidney toxicity. Moreover, our multifunctional nanomedicine conjugates Fe^2+^ ions to EGCG to achieve better ferroptosis-promoting effects through promoting the Fenton reaction by increasing intracellular Fe^2+^ levels. Additionally, Fe-EGCG degrades under low pH conditions, and it is also conducive to encapsulating small-molecule drugs within its structure. This enables tumor-enriched delivery of RSL3, facilitating its release within cancer cell lysosomes. These characteristics enable Fe-EGCG@RSL3 to exert antitumor effects against BCa without significant systemic toxicity. Furthermore, the construction process of our nanomedicine, pH-biased jet mixing of self-small molecules, is straightforward for large-scale production, offering significant potential for clinical translation.

Responsive nanomaterials offer numerous advantages in enhancing the therapeutic efficacy of ferroptosis inducers and providing improved opportunities for ferroptosis-inducing cancer therapy. However, previously reported ferroptosis-inducing nanomaterials warrant assisted physical activation methods to exert best effects, including heating and laser irradiation [30,31]. These lead to constraints in clinical use, especially in the bladder, a deep pelvic organ. Our nanomedicine can effectively accumulate in tumor tissues after administration and spontaneously respond, overcoming the limitations of conventional physical response methods for deep pelvic organs. Regarding the nano vehicles, the commonly used carriers of nanomedicines, including TA, polyvinylpyrrolidone, and Au nanoparticles, only play the role of simple delivery or assisting drug release rather than effectively participating in the induction process of ferroptosis. Hsieh et al. [32] reported an ultrasmall polyvinylpyrrolidone nanomedicine carrying the Fe–Cu–Ni–S complex to induce ferroptosis in cancer. Ma et al. [33] reported that another nanomedicine using TA as a nanocarrier delivers Fe^3+^ and HMME to induce ferroptosis in colorectal cancer. The aforementioned nanomedicines require auxiliary delivery elements to perform ferroptosis effectively in vivo. In the nanomedicine we developed, both components of the nanocarrier, i.e., Fe^2+^ and EGCG, can participate in the process of promoting ferroptosis, which has increased utilization efficiency.

Intravesical instillation is widely used in treating BCa in clinical practice and can effectively increase the effective concentration within the tumor and prevent first-pass elimination during systemic administration [34,35]. We demonstrated that our nanomedicine effectively accumulates in the mouse bladder, suppresses tumor growth, and enhances immune responses during intravesical instillation. These findings highlight the nanomedicine’s multifunctional antitumor efficacy via local administration, in contrast to prior nanomedicine approaches targeting BCa, which have been assessed solely through a single administration method. The dual-route administration assessment not only demonstrated the therapeutic versatility of our nanomedicine but also significantly improved its clinical translation potential.

Ferroptosis has been well characterized as a crucial component of the cytotoxic mechanism mediated by CD8^+^ T cells [36]. Inducing ferroptosis to promote T-cell-related immunotherapy efficacy has been a promising strategy. In the management of pancreatic ductal adenocarcinoma, a dihydroartemisinic-derived nanoparticle conjugated with RSL3 has been reported to be administered in combination with programmed cell death ligand 1 (PD-L1) blockade therapy [37]. However, anti-PD1 therapy is recommended as part of first-line therapy for advanced urothelial carcinoma. Hence, we evaluated the efficacy of Fe-EGCG@RSL3-induced sensitization to anti-PD1 therapy in vivo. Our results indicate that Fe-EGCG@RSL3 is a multifaceted nanoparticle platform that not only directly induces tumor cell ferroptosis but also enhances the efficacy of anti-PD1-based immunotherapy. Furthermore, single-cell transcriptome sequencing was conducted to better elucidate the remodeling of the complex tumor microenvironment by nanomedicine.

Some of the components of the tumor immune microenvironment are altered by not only the administration of Fe-EGCG@RSL3 but also the administration of Fe-EGCG. Specifically, the proportion of naïve T cells decreased, and the number of *Gzmk*-expressing effector memory CD8^+^ T cells increased. These findings indicate the potential of Fe-EGCG to promote the differentiation of naïve T cells into effector memory CD8^+^ T cells. Notably, *GZMK*-expressing CD8^+^ T cells are positively correlated with the response to immunotherapy in clear cell renal cell carcinoma [38], esophageal cancer [37], and peripheral blood of non-small cell lung cancer patients [39]. Thus, Fe-EGCG in our nanomedicine facilitates the differentiation of naïve CD8^+^ T cells into *Gzmk*-expressing CD8^+^ T cells, which may enhance the efficacy of immunotherapy to a certain extent. Furthermore, we observed that the proportion of *Folr2*-expressing macrophages decreased under Fe-EGCG or Fe-EGCG@RSL3 treatment. FOLR2-expressing macrophages have been reported as a subpopulation with controversial functions in the tumor microenvironment. In breast tumors, *FOLR2*-expressing macrophages are correlated with better patient survival [40]. In immunotherapy-related single-cell analyses, *FOLR2*-expressing macrophages are identified primarily as M2-like protumor clusters that restrict immunotherapy efficacy [41,42]. The diminished proportion of M2-like *Folr2*-expressing macrophages may also dictate the enhancement of immunotherapy by our nanomedicine. Collectively, these remodeling effects of the tumor immune microenvironment are potentially due to the intrinsic properties of the nanomedicine, which are independent of the secondary effects associated with tumor ferroptosis.

Furthermore, we reported several unique effects of nanomedicine on microenvironment remodeling, which may be largely due to the derivative influence of the ferroptosis-inducing effect in the TME. First, CD8^+^ T-cell-mediated cytotoxicity has been characterized as a ferroptosis-inducing process [36]. Thus, the combined effects of anti-PD1 immunotherapy-induced ferroptosis and nanomedicine-induced ferroptosis resulted in the best control of tumor progression. Furthermore, neutrophils possess well-known tumor immunosuppressive effects within the immune microenvironment [43]. In our study, neutrophil infiltration was significantly decreased as Fe-EGCG@RSL3 treatment was added to anti-PD1 immunotherapy. In CD4^+^ T cells, Fe-EGCG@RSL3 treatment diminished *Jun/Fos*-expressing stress response state T cells, which has been similarly identified as an immunotherapy resistance-related CD4^+^ T-cell subpopulation in single-cell analyses across 16 types of human cancers [22]. Moreover, *Cxcr3*-expressing Th1-like CD4^+^ T cells have been shown to be induced as nanoparticles that target tumor-draining lymph nodes [44], which is consistent with our nanomedicine that targets the tumor niche. The Th1 proportion is also positively correlated with the response to immunotherapy in various cancer types [45,46]. These findings highlight potential nanomedicine-based therapeutic strategies for targeted cancer therapy, mainly due to the secondary effects of ferroptosis induction, as these alterations are observed only under Fe-EGCG@RSL3 treatment but not under Fe-EGCG treatment.

Overall, single-cell sequencing analyses offer a more nuanced portrayal of the Fe-EGCG@RSL3-modulated TME, facilitating a deeper understanding of the mechanisms underlying the nanomedicine that potentiates immunotherapy for BCa. Therefore, Fe-EGCG@RSL3 has dual functions as a multifaceted nanomedicine capable of triggering ferroptosis in BCa cells and remodeling the TME.

## Conclusion

In conclusion, we designed and characterized a novel self-assembled nanomedicine that encapsulates the ferroptosis inducer RSL3, named Fe-EGCG@RSL3. The nanomedicine has multiple functions, including promoting ferroptosis and remodeling the tumor microenvironment, thereby exerting antitumor effects on BCa. In conclusion, Fe-EGCG@RSL3 is a novel, safe, and unique strategy with great potential for the management of BCa in the future.

## Materials and Methods

### Synthesis of Fe-EGCG and Fe-EGCG@RSL3

A total of 45.84 mg of EGCG (Aladdin, China; E276041) was dissolved in 10 ml of double-distilled H₂O (ddH_2_O). Then, 55.60 mg of FeSO_4_·7H_2_O (Macklin, China; I809844) was dissolved in 30 ml of ddH_2_O. Both solutions were precisely adjusted to pH 6.0. Solutions were added to a T-type confined impingement jet mixer with a flow rate of 5 ml/min at room temperature to synthesize the Fe-EGCG nanomedicine. Fe-EGCG was extracted from the solution via centrifugation at 10,000 rpm for 10 min at 4 °C, followed by 2 washes with 40 ml of ethanol. The freshly prepared Fe-EGCG nanomedicine was fully resuspended in ethanol after 15 min of ultrasonic treatment and preserved at −20 °C. To encapsulate RSL3 in Fe-EGCG, 4 mg of RSL3 was first dissolved in 10 ml of ethanol. The extracted Fe-EGCG was resuspended in the RSL3 solution. The aforementioned solution was mixed at 4 °C for 48 h after ultrasonic treatment for 60 min. The Fe-EGCG@RSL3 nanomedicine was extracted from the solution as previously mentioned. The Fe-EGCG@RSL3 nanomedicine was fully resuspended in ethanol after ultrasonic treatment for 15 min and preserved at −20 °C. The drug loading efficiency (DLE) was calculated as DLE (%) = (*W*_1_ − *W*_0_)/*W*_0_ × 100%, where *W*_0_ is the weight of the Fe-EGCG nanomedicine and *W*_1_ is the weight of the Fe-EGCG@RSL3 nanomedicine.

### Characterization of nanomedicine

The morphological characteristics of the nanomedicine were analyzed using a transmission electron microscope (TEM) model JEM-2100F. FTIR spectra were recorded in the range of 4,000 to 400 cm^−1^ utilizing a Nicolet 7000-C spectrometer. Data visualization was conducted using Origin 2024 software, with reported values representing the mean of 3 independent measurements. XRD analysis was performed with a Bruker D8 Advance instrument. Following data processing with Omnic 8.0, Origin 2024 software was employed for graphical representation.

### Cell culture

Human BCa cell lines T24, SW780, RT112, and UM-UC-3, as well as the human urothelial cell line SV-HUC-1 and the mouse BCa cell line MB49, were procured from the American Type Culture Collection (ATCC; Manassas, VA, USA). T24 cells were cultured in Roswell Park Memorial Institute (RPMI)-1640 medium (Gibco, China) supplemented with 10% fetal bovine serum (FBS) and 1% penicillin/streptomycin (Thermo Fisher, USA). SV-HUC-1 cells were maintained in Ham’s F-12K (Gibco, China) with similar supplements. The remaining cell lines (UM-UC-3, SW780, RT112, and MB49) were cultured in Dulbecco’s modified Eagle’s medium (DMEM; Gibco, China) containing 10% FBS and 1% penicillin/streptomycin. All cell lines were incubated in a humidified environment with 5% CO_2_ at 37 °C, and all tested negative for mycoplasma contamination.

### RNA isolation and qRT-PCR

Total RNA was extracted from the cells using TRIzol reagent (TaKaRa Biotechnology, China) in accordance with the manufacturer’s guidelines. The quality and concentration of the extracted RNA were assessed using a Nanodrop 2000 (Thermo Fisher, USA). Reverse transcription of total RNA was performed with a PrimerScript RT-PCR kit (Vazyme, China). Real-time qPCR was conducted following a standard SYBR Green quantitative PCR kit protocol (Vazyme, China) using a LightCycler 480 real-time instrument (Roche, Germany). GAPDH was utilized as the internal control, and relative mRNA expression levels were calculated using the 2^−ΔΔCt^ method. Specific primers employed in the study are detailed in Table S1.

### In vitro cell uptake assay

T24 cells were treated with 44.6 μg ml^−1^ of Fe-EGCG@RSL3 for 2 h. The tumor cell uptake of the nanomedicine was examined with a TEM from JEM-2100F. To label the nanomedicine with fluorescein-5-carboxamide cadaverine (5-FAM; Macklin, China; 1006585-56-3), 2 μg ml^−1^ 5-FAM was mixed with Fe-EGCG in ethanol for 48 h at 4 °C. 5-FAM-labeled Fe-EGCG was added to T24 cells, which were subsequently incubated for the indicated times. The cells were washed with phosphate-buffered saline (PBS) twice and then stained with Hoechst 33342 (Solarbio, China; B8040). Confocal laser scanning microscopy (Zeiss LSM 710, Germany) was used to capture fluorescence images.

### Cell cytotoxicity assays

The AM/PI assay (SolarBio, China) was used to quantify cell viability. DMSO, 1 μM RSL3, 44.6 μg ml^−1^ Fe-EGCG, and 44.6 μg ml^−1^ Fe-EGCG@RSL3 were added to T24 cells or MB49 cells in 24-well plates. AM/PI staining was performed for 30 min at 37 °C after the medium was removed. The fluorescence images were captured through an inverted fluorescence microscope (Olympus IX83). The mean fluorescence intensity (MFI) was calculated via ImageJ to assess cell viability.

A CCK-8 assay (APExBIO, USA) was used to quantify cell viability [47]. DMSO, 1 μM RSL3, 44.6 μg ml^−1^ Fe-EGCG, or 44.6 μg ml^−1^ Fe-EGCG@RSL3 was added to T24 cells and MB49 cells in 96-well plates for 16 h. Then, the medium was removed, and the CCK-8 assay was performed following the manufacturer’s instructions.

### Flow cytometry

A SYTOX Green kit (KeyGEN BioTECH, China) was used to detect cell death. A C11 BODIPY 581/591 lipid peroxidation probe (Thermo Fisher, USA) was used to detect lipid peroxidation. Both assays were performed according to the manufacturers’ protocols. The T24 and MB49 cell lines were analyzed via flow cytometry (Beckman CytoFLEX, USA).

### Intracellular Fe^2+^ measurement

A FerroOrange fluorescent probe (Servicebio, China) was used according to the manufacturer’s protocol to detect the intracellular Fe^2+^ concentration. T24 cells and MB49 cells were seeded in 24-well plates. The cells were treated with DMSO, 1 μM RSL3, 44.6 μg ml^−1^ Fe-EGCG, or 44.6 μg ml^−1^ Fe-EGCG@RSL3 for 4 h. Afterwards, the medium was removed, and the cells were sequentially washed with PBS and solution buffer. The cells were incubated with 0.5 μl of FerroOrange fluorescent probe dissolved in 0.5 ml of solution buffer at 37 °C for 30 min. After washing with solution buffer, the cells were observed and captured under an inverted fluorescence microscope (Olympus IX83, Japan). The Fe^2+^ fluorescence intensity was quantified with ImageJ.

### RNA extraction and sequencing

T24 and MB49 cells were treated with DMSO, 1 μM RSL3, 44.6 μg ml^−1^ Fe-EGCG, or 44.6 μg ml^−1^ Fe-EGCG@RSL3 for 4 h, and biological replicates (*n* = 2) were collected for RNA extraction via a TRIzol reagent kit as we previously described [48]. RNA sequencing and library construction were performed by Novogene Biotechnology (Guangzhou, China). Ferroptosis inducer genes were screened through FerrDb V2 (http://www.zhounan.org/ferrdb/current/). Gene expression profiles were analyzed via R (4.4.1). GSEA was performed via R (4.4.1).

### BCa organoid culture

The methods used for the construction of human and mouse BCa models are described in the Supplementary Materials. Both human and mouse BCa organoids were seeded in 48-well plates. BCa organoids were separately treated with DMSO, 1 μM RSL3, 44.6 μg ml^−1^ Fe-EGCG, or 44.6 μg ml^−1^ Fe-EGCG@RSL3 for 3 h. The medium was removed after the treatment, followed by 2 washes with PBS. PI staining (ServiceBio, China) was used to detect cytotoxicity according to the manufacturer’s instructions. Fluorescence images were captured via an inverted fluorescence microscope (Olympus IX83, Japan) and quantified via ImageJ.

### Subcutaneous mouse model of BCa

The subcutaneous mouse model of MB49 cells in C57BL/6 mice is described in the Supplementary Materials. All 30 subcutaneous mouse models were randomly separated into 6 groups (*n* = 5), and treatment was started after the average tumor volume was greater than 50 mm^3^. PBS, 10 mg kg^−1^ of RSL3, 76.6 mg kg^−1^ of Fe-EGCG, and 76.6 mg kg^−1^ of Fe-EGCG@RSL3 were injected into the tail vein of the mice every 2 days. Anti-mouse PD1 (10 mg kg^−1^; Selleck, China; A2122) was administered through intraperitoneal injection twice a week. The experimental endpoints were determined on the basis of the tumor volume measurement and ethical considerations. The plants were sacrificed under carbon dioxide anesthesia after 26 days of inoculation. The subcutaneous tumors were dissected for measurement, analysis, single-cell sequencing, fixation with paraformaldehyde, embedding in paraffin wax, and sectioning to obtain the largest slice, followed by H&E staining, IHC, and immunofluorescence. The heart, liver, brain, and kidney were also harvested and dissected for H&E staining.

### Humanized subcutaneous mouse models of BCa

T24 cells were digested with trypsin and suspended in PBS at a concentration of 3 × 10^7^ cells ml^−1^. A total of 100 μl of the cell suspension was injected into the right dorsal subcutaneous tissue of non-obese diabetic–severe combined immunodeficient (NOG-SCID) mice. For T-cell immunity rebuilding, 1 × 10^7^ activated human PBMCs were injected into each mouse through the tail vein (PBMC isolation and expansion details are provided in the Supplementary Materials). All 30 NOG-SCID mice were then randomly separated into 6 groups (*n* = 5). Treatment was started after 1 week of T-cell immunity rebuilding. DMSO, 10 mg kg^−1^ RSL3, 76.6 mg kg^−1^ of Fe-EGCG, and 76.6 mg kg^−1^ of Fe-EGCG@RSL3 were separately injected into the mouse tail vein every 2 days. Anti-human PD1 (10 mg kg^−1^; BeiGene, China) was given through intraperitoneal injection twice a week. The plants were sacrificed under carbon dioxide anesthesia after 24 days of inoculation. The subcutaneous tumors were dissected for measurement and analysis, fixed with paraformaldehyde, embedded in paraffin wax, and sectioned to obtain the largest slice, followed by H&E staining, IHC, and immunofluorescence.

### Orthotopic murine models of BCa

MB49 cells transfected with firefly luciferase were digested with trypsin and suspended in PBS at a concentration of 2 × 10^6^ cells ml^−1^. A total of 50 μl of cell suspension was implanted into the bladders of C57BL/6 mice via intravesical instillation to construct an orthotopic murine model of BCa. An IVIS (Tannon, China) was used to identify tumor generation in vivo*.* A total of 30 mice were randomly separated into 4 groups (*n* = 4). The construction of the orthotopic model was confirmed via IVIS 6 days after inoculation. Treatment was started after the average tumor flux reached 1 × 10^6^ [p/s/cm^2^/sr]. PBS, 10 mg kg^−1^ RSL3, 76.6 mg kg^−1^ Fe-EGCG, and 76.6 mg kg^−1^ Fe-EGCG@RSL3 were administered via intravesical instillation every 2 days. Anti-mouse Pd1 (Sellcek, China; A2122) was given through intraperitoneal injection every 2 days. The experimental endpoint is reached when the average fluorescence intensity of the tumors in mice, as shown by in vivo imaging, exceeds 1 × 10^9^ [p/s/cm^2^/sr]*.* Fifteen days after inoculation, all the mice were sacrificed under carbon dioxide anesthesia. Bladders were then dissected for measurement, analysis, fixation with paraformaldehyde, embedding in paraffin wax, and sectioning to obtain the largest slice, followed by H&E staining.

### In vivo biodistribution

To investigate the distribution of the nanomedicine in vivo, 20 μg ml^−1^ DiR (Aladdin, China; D131031) was mixed with Fe-EGCG in ethanol for 48 h at 4 °C to label the nanomedicine. A total of 2 μg ml^−1^ DiR or 100 μl of free DiR or Fe-EGCG-encapsulated equivalent DiR was injected into 2 groups of C57BL/6 subcutaneous tumor-bearing mice (*n* = 3) through intravenous injection. All the mice were anesthetized and photographed at 0, 24, 48, and 72 h via IVIS. All the mice were sacrificed and dissected at 72 h after injection. The tumors and major organs were separated and imaged using a Tanon system. A Tanon system was also used to measure the fluorescence intensity.

### Immunohistochemistry

Immunohistochemical analysis was performed as previously described [49]. Following dewaxing, antigen retrieval, catalase blocking, and general blocking, tissue samples were incubated overnight at 4 °C with rabbit anti-4HNE (R&D, China; MAB3249; 1:400). Subsequent steps included washing, secondary antibody incubation, and 3,3'-diaminobenzidine (DAB) staining. The quantification of 4HNE-positive cells was conducted using ImageJ, calculating the percentage of positive signals in each field of view across the tissues. At least 5 fields per section were analyzed for quantification, and the accuracy of automated measurements was validated by 2 pathologists. Staining intensity was categorized as follows: 1 (0% to 10% staining area), 2 (10% to 30%), 3 (30% to 50%), and 4 (50% to 100%). The 4HNE staining score was computed as the product of intensity and the percentage of stained cells (4HNE staining score = intensity × percentage of positive field of view).

### Immunofluorescence staining

Immunofluorescence staining was conducted as previously described [50]. Antigen retrieval was performed via target retrieval solution, pH 9.0, in a pressure cooker for 15 to 20 min. The fixed tissues were then washed with PBS and permeabilized with 0.2% Triton X-100 in PBS for 20 min. The tissues were blocked in PBS with 2% BSA for 30 min at room temperature. The samples were subsequently incubated with rabbit anti-Tcf1 (CST, USA; 2203T; 1:200), rabbit anti-Gzmb (CST, USA; 46890T; 1:2,000), rabbit anti-mouse Cd8 (CST, USA; 98941; 1:800), rabbit anti-F4/80 (CST, USA; 30325T; 1:1,600), rabbit anti-Cd206 (CST, USA; 24595T; 1:800), rabbit anti-S100a8 (CST, USA; 33254S; 1:5,000), and rabbit anti-Gzmk (Thermo Fisher, USA; PA5-50980; 1:2,000) at 4 °C overnight. After the antibodies mentioned were removed and washed, the tissues were incubated with a tyramide signal amplification assay (Thermo Fisher, USA) according to the manufacturer’s protocol. DAPI (4′,6-diamidino-2-phenylindole) was used to counterstain the nuclei. A multicolor fluorescence quantitative analyzer for whole tissue sections (Akoya Vectra Polaris, USA) was used to obtain the images. For quantification, the cells in at least 5 fields per section were counted. To avoid evaluation bias, the automatic analysis was independently performed by 2 pathologists.

### Single-cell RNA sequencing

The scRNA-seq experiment was executed by personnel at the NovelBio laboratory (China). Libraries for scRNA-seq were generated using the 10X Genomics Chromium Controller and Chromium Single Cell 3′ V3 Reagent Kits (10X Genomics, CA). The amplified barcoded cDNA underwent fragmentation, A-tailing, adaptor ligation, and index PCR amplification. The final libraries were quantified using the Qubit high-sensitivity DNA assay (Thermo Fisher, USA), and their size distribution was analyzed with a high-sensitivity DNA chip on a Bioanalyzer 2200 (Agilent). Sequencing of all libraries was performed on an Illumina sequencer (Illumina, CA) utilizing a 150-bp paired-end run. Data cleaning was achieved using fastp with default parameters to filter adaptor sequences and eliminate low-quality reads. Feature-barcode matrices were generated by aligning reads to the human genome (GRCh38 Ensemble: version 104) using CellRanger v8.0.0. Data normalization, dimensionality reduction, and clustering were conducted using the Seurat v5.2.1 method in R (v4.4.1).

### Statistical analysis

All statistical graphs and analyses were generated using GraphPad Prism 10.0 software, with error bars representing standard error of the mean (SEM) or standard deviation (SD). The statistical significance of differences between groups was assessed using unpaired or paired Student’s *t* tests, one-way analysis of variance (ANOVA), or 2-way ANOVA. Additional statistical analyses were conducted using R (v4.4.1).

## Data Availability

Data generated during the current study are available from the corresponding authors upon reasonable request.
