# Supplementary material for: A Multifunctional Fe-EGCG@RSL3 Nanomedicine Synergizes Ferroptosis Induction and Tumor Microenvironment Remodeling for Enhanced Bladder Cancer Immunotherapy
Source: Research (Wash D C). 2025 Jun 17;8:0735. doi: 10.34133/research.0735 (PMC12173478; doi:10.34133/research.0735)
Supplement: Supplementary 1 — Supplementary Methods Figs. S1 to S21 Table S1 [file research.0735.f1.docx]

**Supplementary Matirials**

**A multifunctional Fe-EGCG@RSL3 nanodelivery system synergizes ferroptosis induction and tumor microenvironment remodeling for enhanced bladder cancer immunotherapy**

Chengjunyu^1,2,5^ Zhang, Sen Liu^1,2,5^, Jianhui Zhang^1,2,5^, Junlin Lu, ^1,2^ Zehua Chen^1,2^, Bolin Pan^1,2^, Chu Liu^1,2^, Ming Huang^1,2^, Hengji Zhan^1,2^, Hongjin Wang^1,2^, Siting Chen^1,2^, Kaiwen Jie^1,2^, Baoqing He^1,2^, Jingdie Wu^1,2^, Ye li^1,2^, Haifeng Wang^3^, Jing Zhao^4,^ *, Qiang Zhang^1,2,^ *, Xu Chen^1,2,^ *

1. Department of Urology, Sun Yat-sen Memorial Hospital, Sun Yat-sen University, Guangzhou, China.

2. Guangdong Provincial Key Laboratory of Malignant Tumor Epigenetics and Gene Regulation, Sun Yat-Sen Memorial Hospital, Sun Yat-Sen University, Guangzhou, China.

3. Department of Urology, The Second Affiliated Hospital of Kunming Medical University, Kunming, China.

4. Scientific Research Center, The Seventh Affiliated Hospital of Sun Yat-Sen University, Shenzhen, China.

5. These authors contributed equally.

*Corresponding authors contribute equally:

Jing Zhao: [zhaoj265@mail.sysu.edu.cn](mailto:zhaoj265@mail.sysu.edu.cn)

Qiang Zhang: [zhangq399@mail.sysu.edu.cn](mailto:zhangq399@mail.sysu.edu.cn)

Xu Chen: [chenx457@mail.sysu.edu.cn](mailto:chenx457@mail.sysu.edu.cn)

**Methods**

***Hemolysis test of Fe-EGCG@RSL3***

The blood samples were obtained from patients at Sun Yat-sen Memorial Hospital of Sun Yat-sen University. Peripheral blood mononuclear cells (PBMCs) were removed using a lymphocyte separation medium. The erythrocyte pellet at the bottom was collected, and 5 mL of 0.9% sodium chloride solution was added. The mixture was centrifuged at 1400 rpm for 10 minutes. After discarding the supernatant, the above steps were repeated until the supernatant became colorless and resuspended to 2% (v/v). The Fe-EGCG@RSL3 solution was prepared using 0.9% sodium chloride solution to achieve final concentrations of 44.6 μg/mL, 22.3 μg/mL, 11.2 μg/mL, 5.6 μg/mL, and 2.8 μg/mL and were mixed 1:1 with the erythrocyte suspension, alongside negative (0.9% NaCl) and positive (double distilled water) controls, followed by 1-hour incubation at 37°C. An inverted microscope (Olympus IX83) was used to photograph the morphology of erythrocytes after co-incubation. The hemolysis rate was analyzed with ImageJ. The co-incubated liquid was centrifuged under the conditions described above, and the hemolysis status was then photographed.

***ROS level measurement of polyphenol and Fe-EGCG@RSL3***

For ROS level detection of the 6 polyphenol we mentioned, DMSO, EGCG (20 μM), Curcumin (20 μM), Rhein (20 μM), Tannic acid (TA, 20 μM), Gallic acid (20 μM) and caffeic acid (CA, 20 μM) were added into T24 and MB49 cells for 1 hour. For ROS level detection of nanomedicine, DMSO, RSL3 (1 μM), Fe-EGCG (22.3 μg/mL) and Fe-EGCG@RSL3 (22.3 μg/mL) were added into T24 and MB49 cells for 1 hour. After the cells were digested and washed for twice,5μM of DCFH-DA (MCE, China; HY-D0940) were added into them and were incubated at 37℃ for 15min avoiding light then washed with PBS twice. Flow cytometry was used to detect the change of ROS level. Flow Jo V10 was used to analyze the relative ROS level.

***Bladder cancer organoid construction and culture***

Newly removed human bladder cancer tumors were separated from human patients derived from Sun Yat-sen Memorial Hospital, Sun Yat-sen University (Guangzhou) and were washed three times in PBS containing 50 μg/mL nystatin, 500 μg/mL streptomycin and 500 U penicillin. The samples were subsequently cut into small parts and digested with collagenase II and DNase I at 37°C for 15 minutes. After digestion, the suspension was passed through a 70 μm cell strainer to remove undigested tissues. The collected cells were counted and resuspended in complete culture media mixed with a 1:4 volume of Matrigel (Corning, USA) at a concentration of 20,000 cells/30 μL. The cell suspensions were plated in a 48-well plate with 30 μL droplets per well. Complete medium for bladder cancer organoids was added after the Matrigel was polymerized. Mouse bladder cancer organoids were a gift from Professor Wei Qiang (Department of Urology, West China Hospital). Bladder cancer organoids were then cultured in an atmosphere of 5% CO_2_ at 37°C.

***Western Blot***

The Western Blot assay was mentioned before (36670069). Briefly, T24 and MB49 cells were treated with DMSO and Fe-EGCG@RSL3 (33.5 μg/mL) for 4 hours. Proteins were extracted and the concentration was measured. Total proteins were separated using SDS-PAGE on 10% gels and transferred to 0.45 μm polyvinylidene fluoride (PVDF) membranes. After block of the membranes, anti-GAPDH (Proteintech, China; 60004-1-Ig; 1:10000), anti-ATF3 (Abclonal, China; A1852; 1:1000), anti-HO1 (Proteintech, China; 10701-1-AP; 1:1000), anti-CHAC1 (Proteintech, China; 15207-1-AP;1:1000) and anti-PTGS2(Proteintech, China; 27308-1-AP; 1:1000) were separately incubated with the membranes at 4℃ overnight. After washing by tris buffer saline with Tween-20 (TBST), the membranes were then incubated with HRP conjugated goat-anti-rabbit secondary antibodies (1:10000) accordingly and visualized using enhanced chemiluminescence.

***Subcutaneous mouse model construction***

Subcutaneous mouse model was conducted as previously described [40]. MB49 cells were digested with trypsin and suspended in PBS at a concentration of 3x10^6^ cells/mL. A total volume of 100 μL of the cell suspension was injected into the right dorsal subcutaneous tissue of C57BL/6 mice. The length and width were measured with a Vernier caliper. The tumor volume (mm^3^) was calculated as length (mm) × width (mm) × width (mm) × 0.5.

***Human primary T cells isolation and activation***

Human primary T cells were isolated from human peripheral blood mononuclear cells derived from Sun Yat-sen Memorial Hospital, Sun Yat-sen University (Guangzhou, China), and were then cultured in RPMI-1640 supplemented with 10% FBS and 30 units mL^-1^ IL-2 (APExBIO, USA; P1020--05) for activation. Human T cells were expanded with Dynabeads Human T-Activator CD3/CD28 (Gibco, USA; 11163D). Dynabeads were added at a bead-to-cell ratio of 1:1. The beads were separated with a magnet after 3 days of coincubation, and the cells were immediately prepared for animal assays.

***Flow cytometry T-cell infiltration and exhaustion detection***

For T-cell infiltration and exhaustion detection, the subcutaneous tumors were dissected and washed with PBS twice. Then, the tumors were cut into small parts and digested with collagenase II and DNase I at 37°C for 30 minutes. After digestion, the suspension was passed through a 70 μm cell strainer to remove undigested tissues. Five milliliters of diluted erythrocyte lysis buffer (Miltenyi, China; 130--094--183) was added at room temperature for 5 min to eliminate erythrocytes. After being washed with PBS, the cells were counted with an automatic cell counter (Alit Biotech, China), and the concentration of the cell suspension was adjusted to 1×10^6^ cells mL^-1^. Ghost Dye (CST, USA; 18452S; 1:1000) was used at room temperature in the dark for 10 minutes to differentiate living and dead cells. For T-cell infiltration and exhaustion detection, fluorescence-labeled antibodies, including anti-mouse Cd45 (Thermo Fisher, USA; MCD4501; 1:1000), anti-mouse Cd11b (Thermo Fisher, USA; A15390; 1:1000), anti-mouse Cd8 (MHCD0804 1:2000), and anti-mouse Pd1 (BioLegend, USA; 29F.1A12; 1:1000), were added, and the samples were incubated at room temperature in the dark for 30 minutes. T-cell infiltration and exhaustion were detected through flow cytometry. The flow gating strategy is mentioned in Fig. S21.


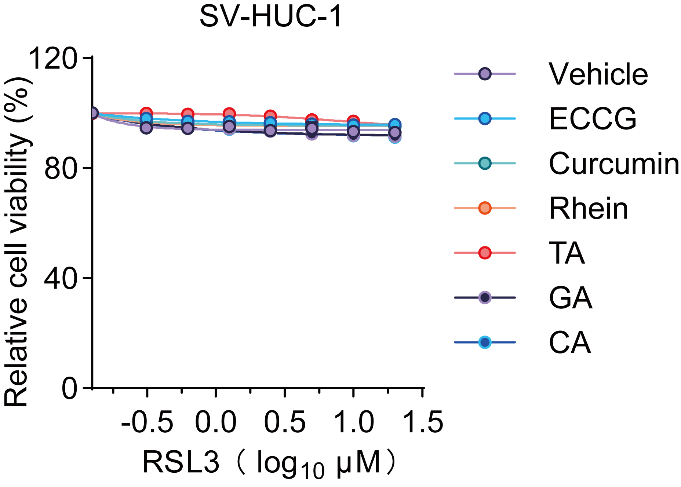


**Figure S1.** Cell viability of urothelium cell line SV-HUC-1 treated with RSL3 along with vehicle, EGCG, Curcumin, Rhein, TA, GA or CA for 16 hours.


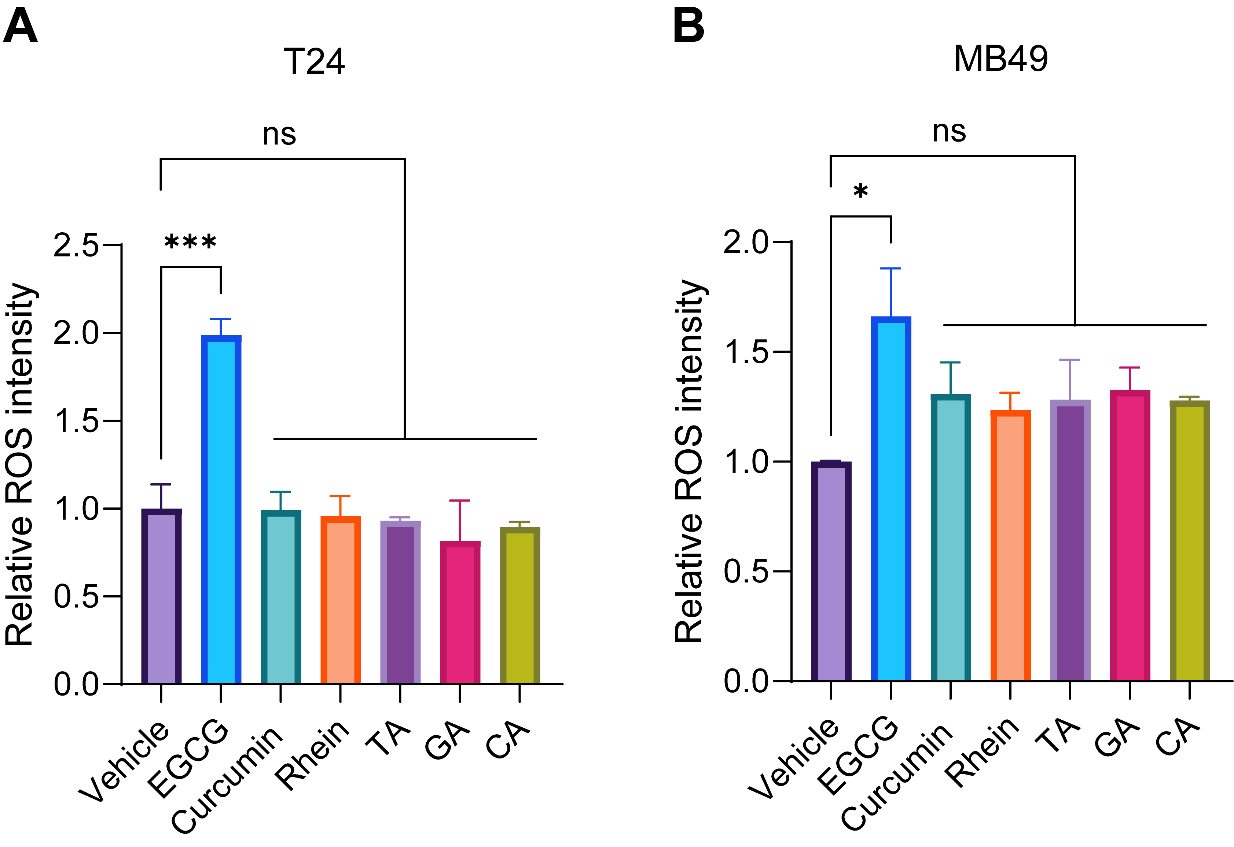


**Fig. S2. EGCG exhibited the strongest ROS-inducing effect in both T24 and MB49 cells.** Relative intracellular ROS levels measured by DCFH-DA in T24 (a) and MB49 (b) cells treated with DMSO, EGCG (20 μM), curcumin (20 μM), rhein (20 μM), tannic acid (TA, 20 μM), gallic acid (20 μM) or caffeic acid (CA, 20 μM) for 1 hour. The data are presented as the means ± SDs. Statistical significance was assessed via one-way ANOVA (ns, P > 0.05; *, P < 0.05; ***, P < 0.001).


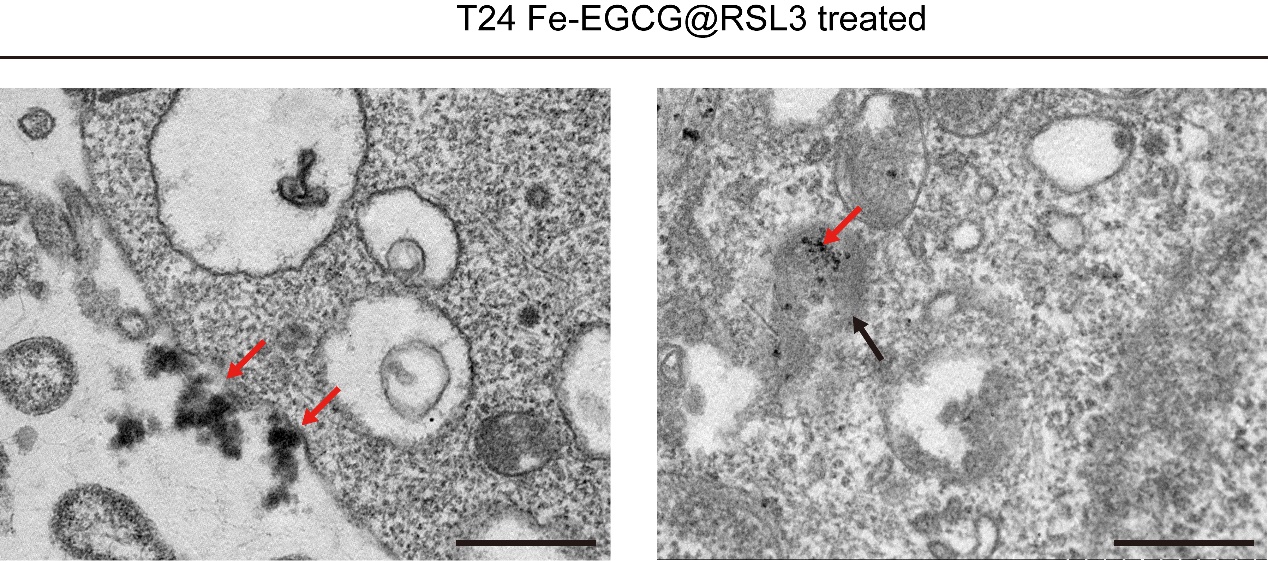


**Figure S3**. TEM images of Fe-EGCG@RSL3 treated T24. Arrows indicated the uptake of Fe-EGCG@RSL3 (Red) into the lysosomes (black) and depolymerized in T24 cells. Scale bar: 500nm.


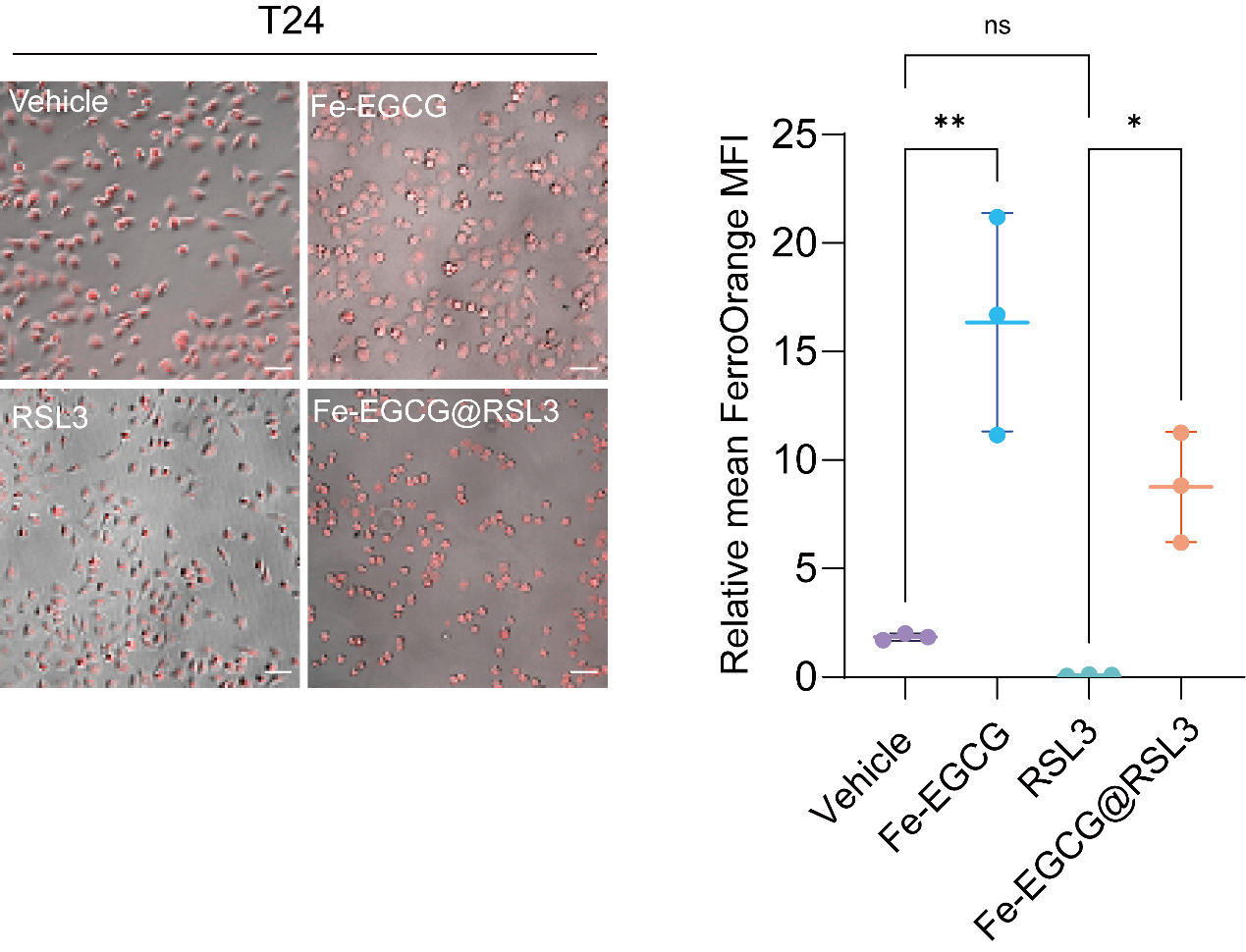


**Figure S4.** Fluorescence images showing Fe^2+^ uptake in T24 cells after treatment with DMSO, RSL3 (1 μM), Fe-EGCG (46.6 μg mL^-1^) or Fe-EGCG@RSL3 (46.6 μg mL^-1^) for 3 hours. Scale bar: 50μm. The data are presented as the means ± SDs. Statistical significance was assessed via one-way ANOVA (ns, *P* > 0.05; *, *P* < 0.05; **, *P* < 0.01).


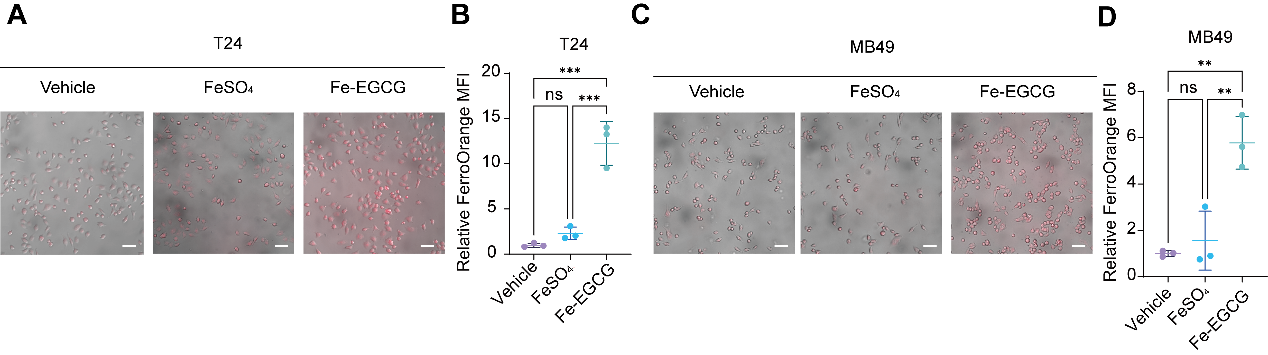


**Fig.S5.** Fluorescence images showing Fe2^+^ uptake after treatment with Vehicle, FeSO_4_ (30 μM) or Fe-EGCG (45 μg mL^-1^) in T24(A, B) and MB49 (C, D) cells for 2 hours. Scale bar: 20 μm. The data are presented as the means ± SDs. Statistical significance was assessed via one-way ANOVA (ns, P > 0.05; **, P < 0.01; ***, P < 0.001).


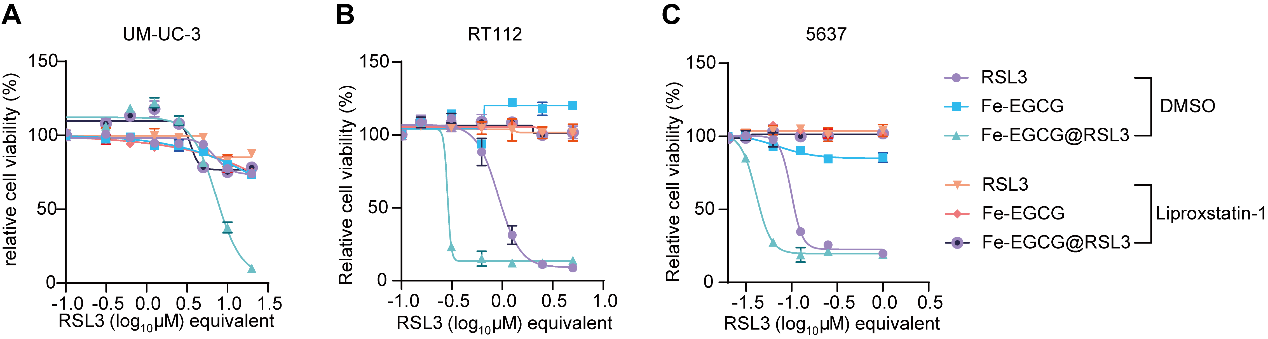


**Fig. S6. Fe-EGCG@RSL3 induces ferroptosis in various bladder cancer cells.** Cell viability of UM-UC-3 (A), RT112 (B) and 5637 (C) cells treated with RSL3, Fe-EGCG and Fe-EGCG@RSL3 (along with DMSO or liproxstatin-1) for 16 hours.


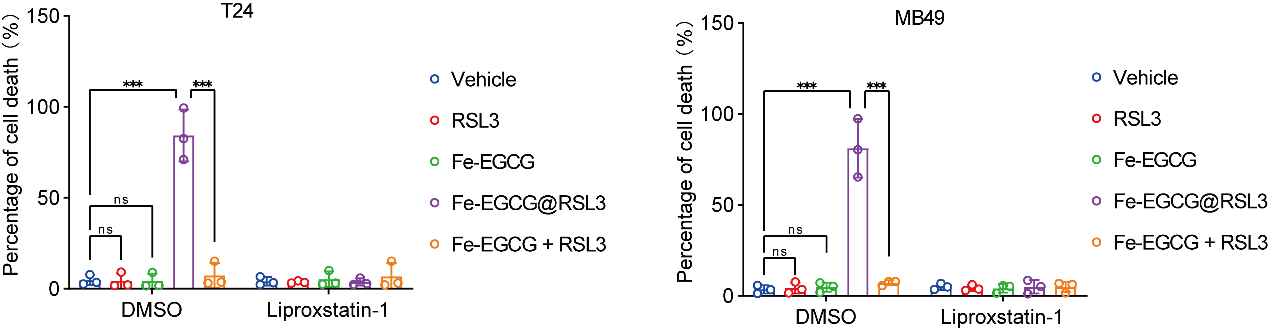


**Figure S7.** Cell death of T24 (left panel) and MB49 (right panel) cells treated with RSL3 (1 μM), Fe-EGCG (44.6 μg mL^-1^), Fe-EGCG@RSL3 (44.6 μg mL^-1^) and Fe-EGCG + RSL3 via SYTOX green staining analyzed by flow cytometry. The data are presented as the means ± SDs. Statistical significance was assessed via one-way ANOVA (ns, *P* > 0.05; ***, *P* < 0.001).


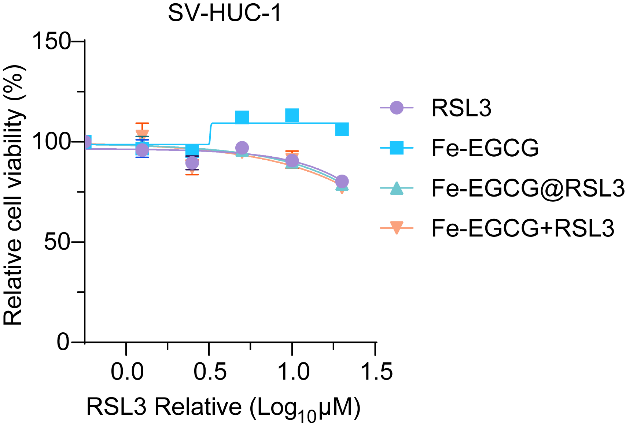


**Fig. S8.** Cell viability of SV-HUC-1 cells treated with RSL3, Fe-EGCG, Fe-EGCG@RSL3, and Fe-EGCG along with RSL3 for 16 hours.


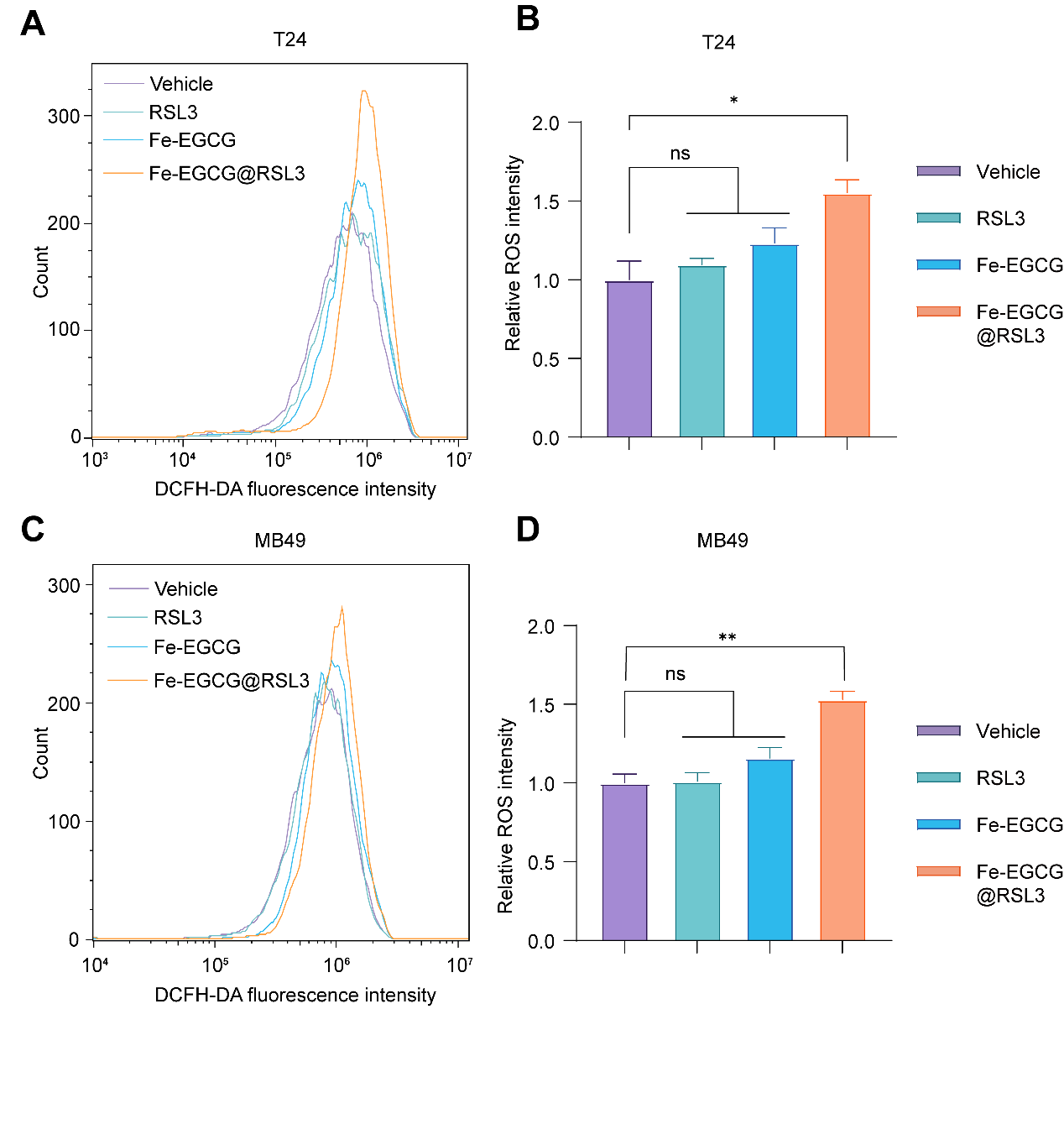


**Fig.S9.** Relative intracellular ROS level measured by DCFH-DA inT24 (A, B) and MB49 (C, D) cells treated with DMSO, RSL3(1 μM), Fe-EGCG (22.3 μg/mL and Fe-EGCG @RSL3 (22.3 μg/mL) for 1 hour. The data are presented as the means ± SDs. Statistical significance was assessed via one-way ANOVA (ns, P > 0.05; *, P < 0.05; ***, P < 0.001).


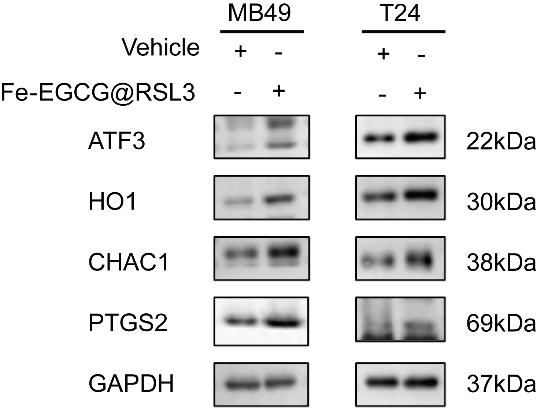


**Fig.S10.** Immunoblot analysis of ATF3, HO1, CHAC1, PTGS2 and GAPDH in MB49 and T24 cells after PBS or Fe-EGCG@RSL3 treated for 4 hours.


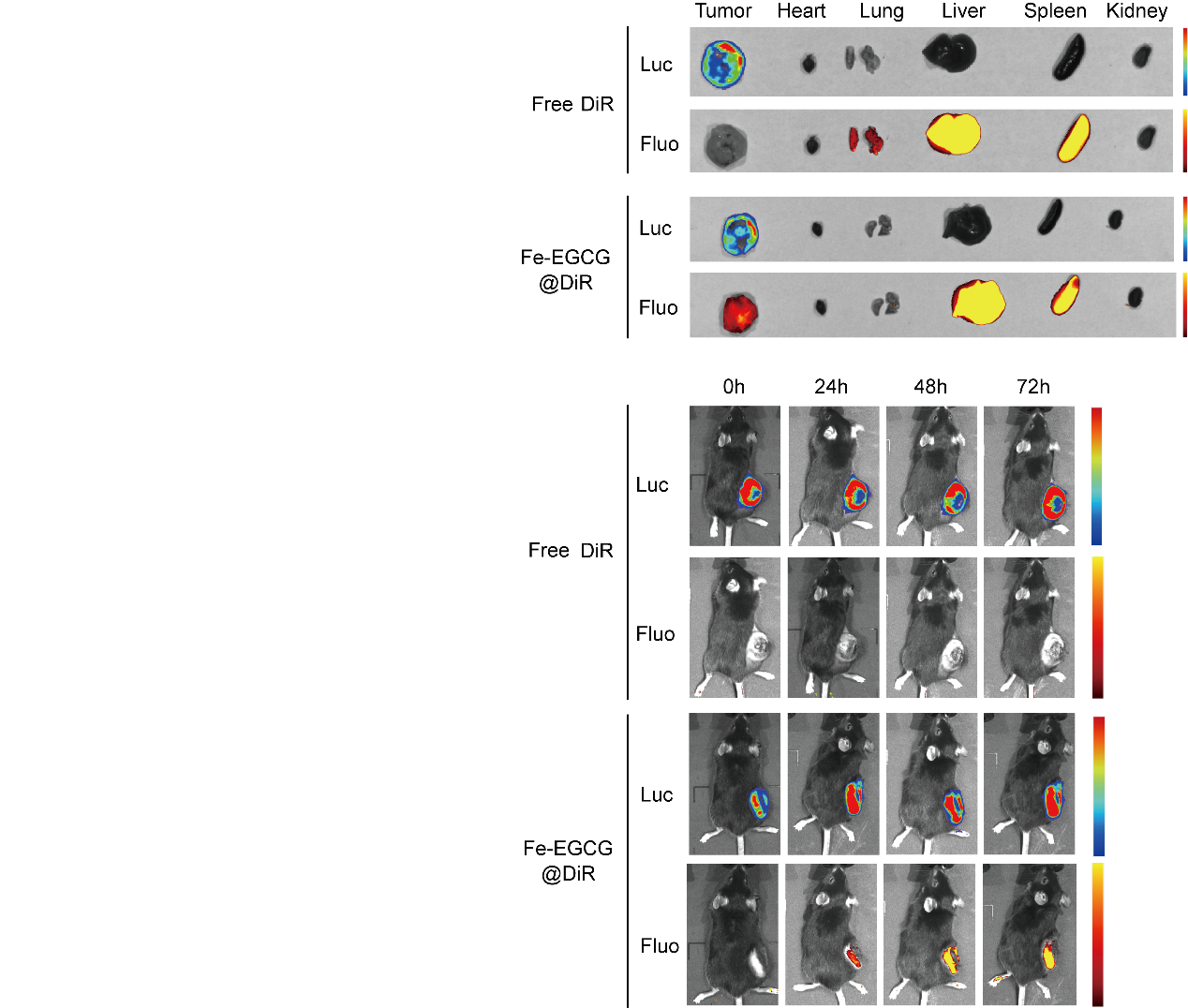


**Figure S11.** Nanomedicine distribution *in vivo*. In subcutaneous mouse bladder cancer model, free DiR or Fe-EGCG@DiR is administered via tail vein injection. Fluorescence and bioluminescence signal is adopted from different organs *ex vivo* (upper panel), and across indicated times *in vivo* (lower panel).


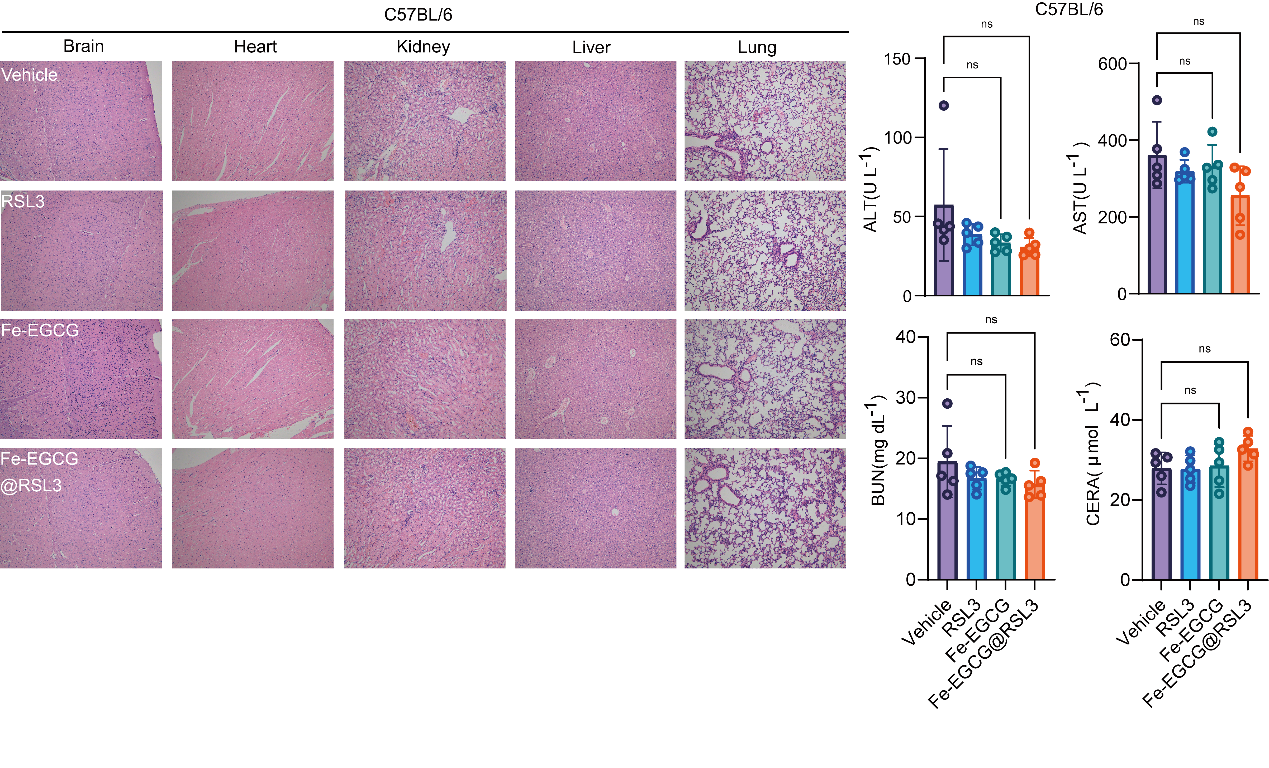


**Figure S12.** Safety profiles of nanomedicine treated C57BL/6 subcutaneous tumor bearing mice. H&E staining images of essential organs in different groups (left panel). Serum biochemistry test involving ALT, AST, CRE, UREA in different groups (right panel). The data are presented as the means ± SDs. Statistical significance was assessed via one-way ANOVA. (ns, *P* > 0.05)


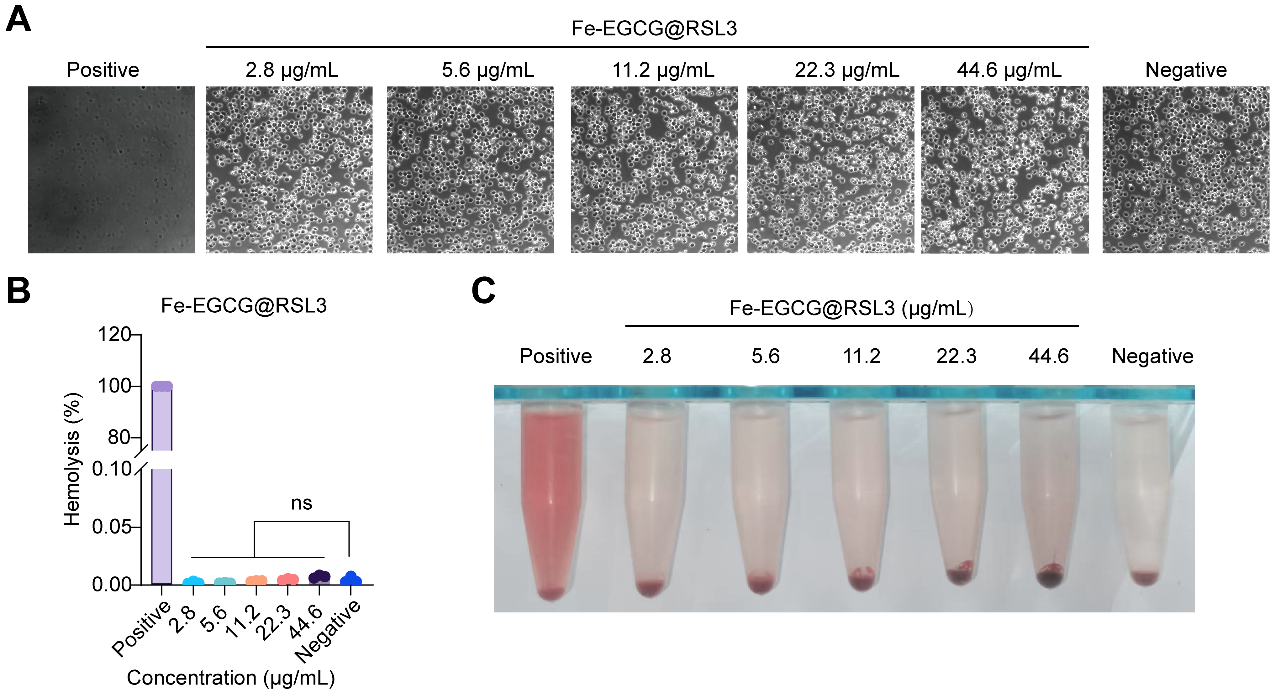


**Fig. S13. Fe-EGCG@RSL3 causes little significant damage to circulating red blood cells.** Microscope photography (A) and statistical analysis (B) of the hemolysis ratio of Fe-EGCG@ESL3 -treated red blood cells (44.6 μg/mL, 22.3 μg/mL, 11.2 μg/mL, 5.6μg/mL and 2.8 μg/mL) and macroscopic photography (C) of red blood cells treated with the indicated concentrations of Fe-EGCG@RSL3. The positive control group was treated with ddH_2_O, and the negative control group was treated with 0.9% NaCl solution. The data are presented as the means ± SDs. Statistical significance was assessed via one-way ANOVA (***, P < 0.001, ***, P < 0.0001).


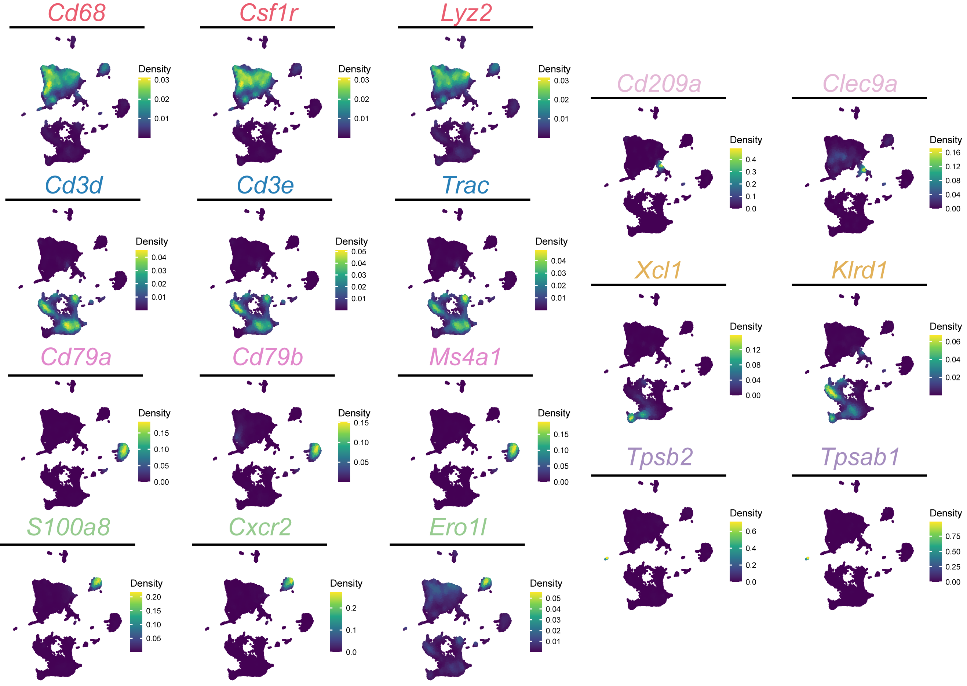


**Figure S14.** Density plots of feature markers expression for myeloid cells, T cells, B cells, neutrophils, DC cells, NK cells and mast cells.


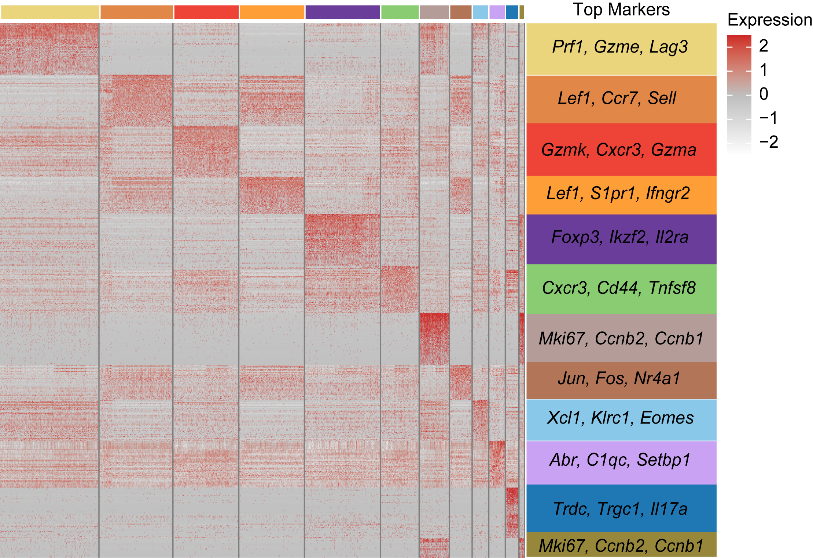


**Figure S15.** Heatmap for top 50 high expressed marker genes of each T cell cluster.


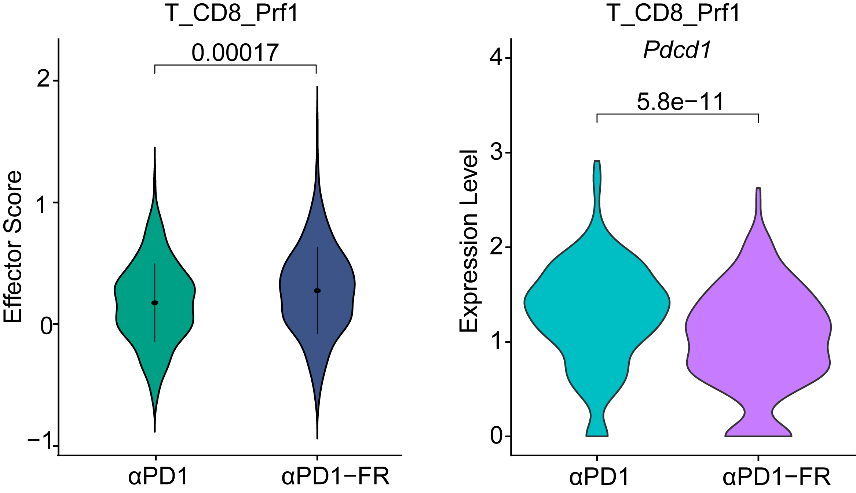


**Figure S16.** Effector gene scores (left panel) and *Pdcd1* expression levels (right panel) of cells from cluster T_CD8_Prf1.


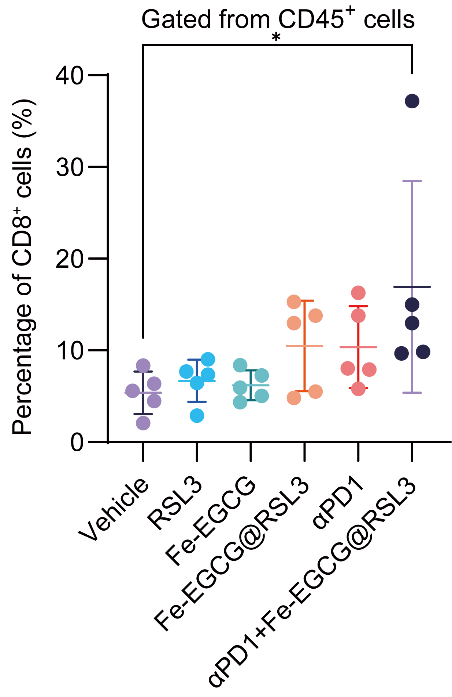


**Figure S17.** Flow cytometry analysis of CD8 T cells from harvested tumors in subcutaneous tumor model of C57BL/6 mice. The data are presented as the means ± SDs. Statistical significance was assessed via one-way ANOVA (ns, *P* > 0.05).


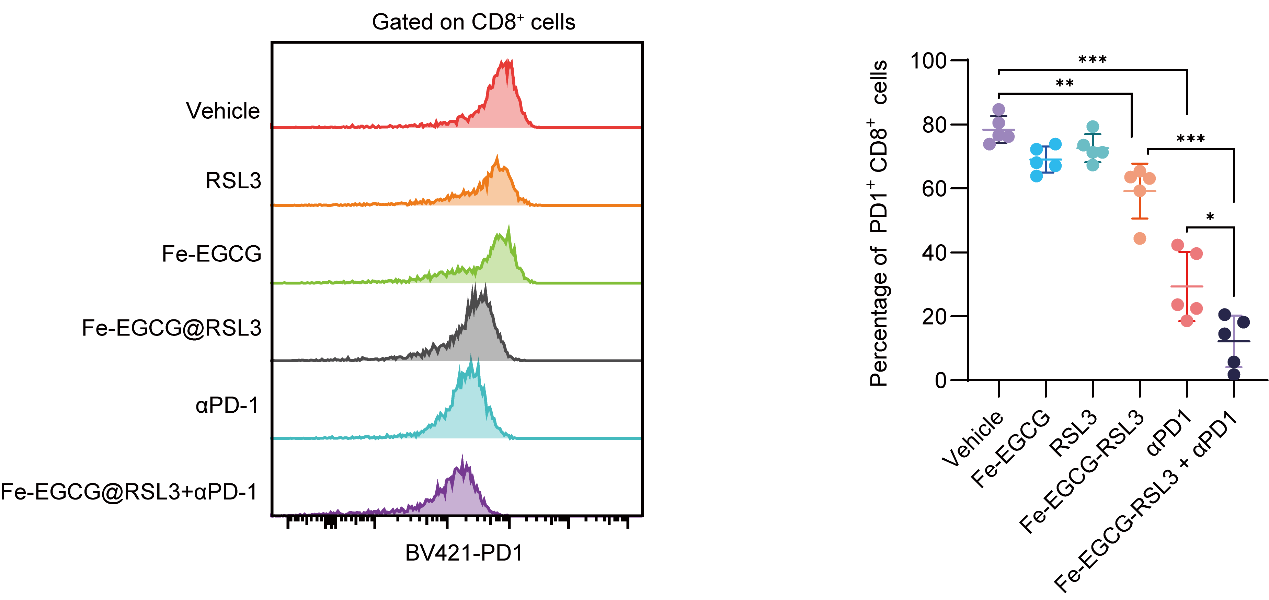


**Figure S18.** Representative flow cytometry images and quantitative analysis of PD1 expression of CD8^+^ T cells in MB49 tumors.


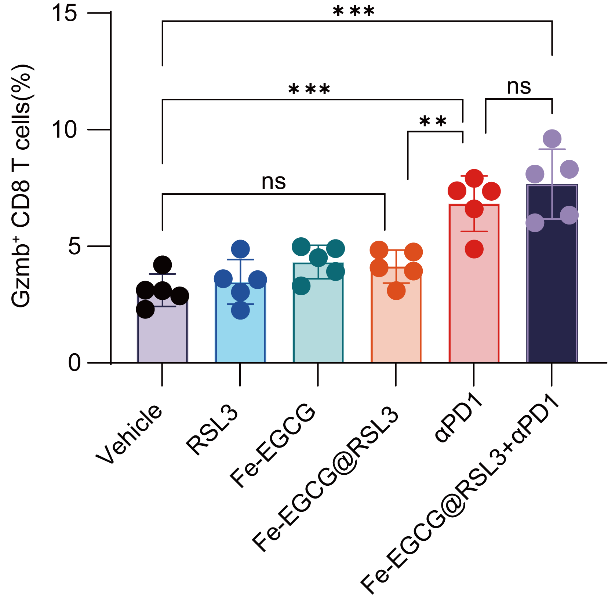


**Figure S19.** Quantitative analysis of Gzmb^+^ CD8^+^ T cells of immunofluorescence images from different groups. Data are presented as means ± SD. Statistical significance was assessed using ANOVA (ns, *P* > 0.05; **, *P* < 0.01; *** *P* < 0.001).


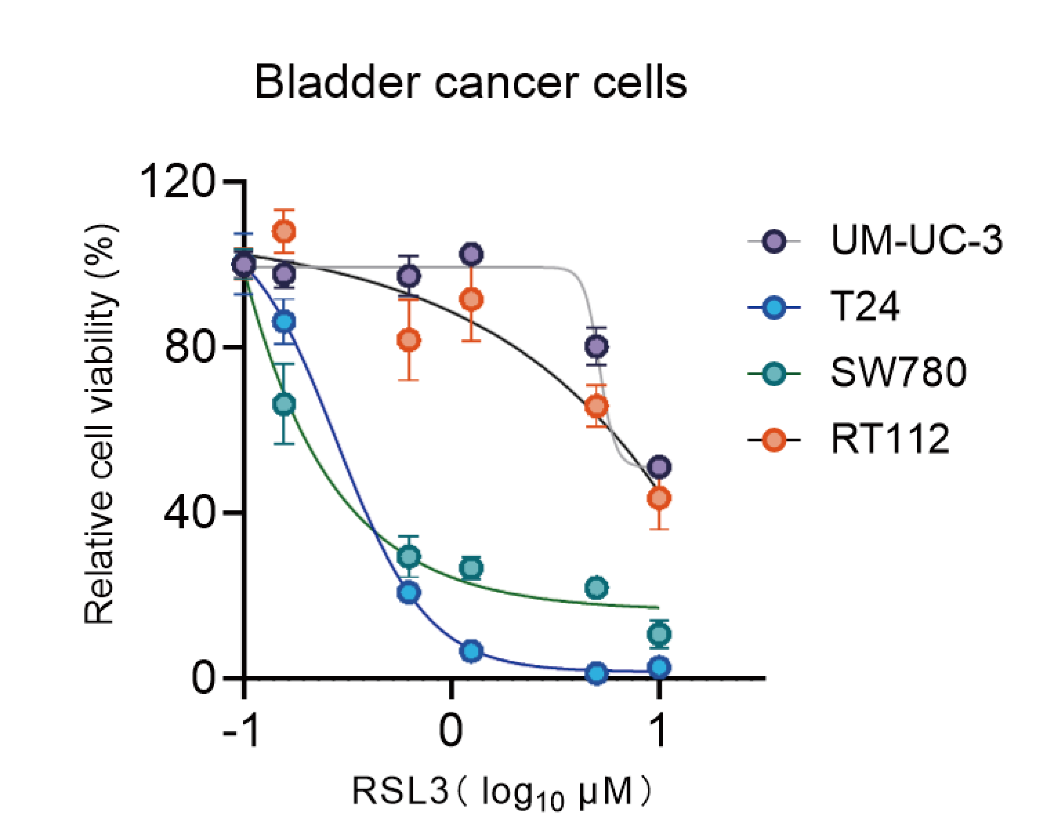


**Figure S20.** Cell viability in different bladder cancer cell lines treated by RSL3.


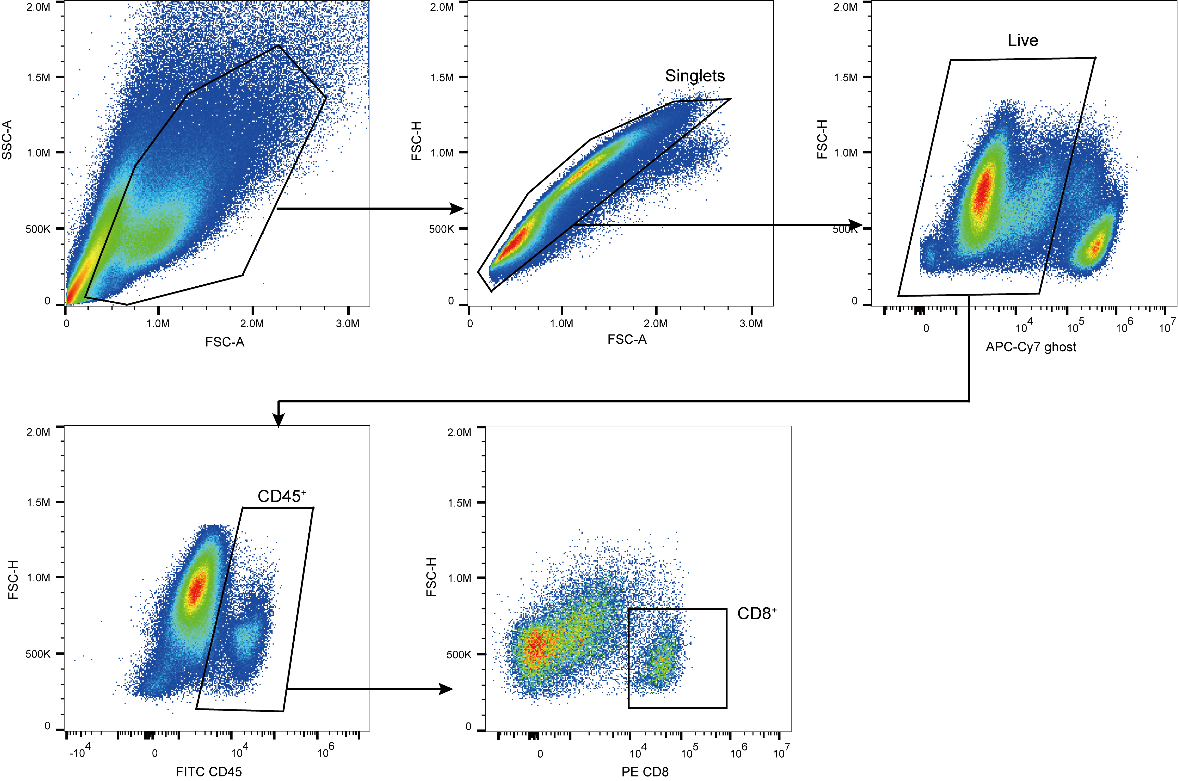


**Fig. S21** Gating strategy of flow cytometry analyzing CD8^+^ T cells from harvested tumors in C57BL/6 subcutaneous mouse model.

**Table S1. RT-qPCR primers used in this study**

| name | sequence:5’-3’ |
| --- | --- |
| HOMO-GAPDH-F | GTCTCCTCTGACTTCAACAGCG |
| HOMO-GAPDH-R | ACCACCCTGTTGCTGTAGCCAA |
| homo-HMOX1-F | CCAGGCAGAGAATGCTGAGTTC |
| homo-HMOX1-R | AAGACTGGGCTCTCCTTGTTGC |
| homo-ATF3-F | CGCTGGAATCAGTCACTGTCAG |
| homo-ATF3-R | CTTGTTTCGGCACTTTGCAGCTG |
| homo-CHAC1-F | GTGGTGACGCTCCTTGAAGATC |
| homo-CHAC1-R | GAAGGTGACCTCCTTGGTATCG |
| homo-PTGS2-F | CGGTGAAACTCTGGCTAGACAG |
| homo-PTGS2-R | GCAAACCGTAGATGCTCAGGGA |
| MUS-GAPDH-f | CATCACTGCCACCCAGAAGACTG |
| MUS-GAPDH-r | ATGCCAGTGAGCTTCCCGTTCAG |
| mus-Atf3-F | GAAGATGAGAGGAAAAGGAGGCG |
| mus-Atf3-R | GCTCAGCATTCACACTCTCCAG |
| mus-Chac1-F | TGACCCTCCTTGAAGACCGTGA |
| mus-Chac1-R | AGTGTCATAGCCACCAAGCACG |
| mus-Hmox1-F | CACTCTGGAGATGACACCTGAG |
| mus-Hmox1-R | GTGTTCCTCTGTCAGCATCACC |
| mus-Ptgs2-F | GCGACATACTCAAGCAGGAGCA |
| mus-Ptgs2-R | AGTGGTAACCGCTCAGGTGTTG |
